# Supplementary material for: Comparative connectomics of dauer reveals developmental plasticity
Source: Nat Commun. 2024 Feb 27;15:1546. doi: 10.1038/s41467-024-45943-3 (PMC10899629; doi:10.1038/s41467-024-45943-3)
Supplement: Supplementary file 1 — Supplementary Information [file 41467_2024_45943_MOESM1_ESM.pdf]

Supplementary Information

Yim, Choe, Bae et al.

Comparative connectomics of dauer reveals developmental plasticity

Supplementary Figures

Supplementary Fig. 1. Reconstruction pipeline and comparison with other datasets.

Supplementary Fig. 2 Morphological changes of neurons in the dauer stage.

Supplementary Fig. 3 Connection properties across development.

Supplementary Fig. 4 Morphological changes are accompanied by new synaptic inputs and outputs.

Supplementary Fig. 5. Connectivity changes in dauer neurons are correlated with unique behavior in dauer.

Supplementary Fig. 6. Other basic network properties.

Supplementary Fig. 7. Type-wise results on out- and in-degree, and whole connectome-wise comparison of dauer and adults.

Supplementary Fig. 8. Expanded and type-wise version of the number of common connections and connection similarity in dauer and adult networks.

Supplementary Fig. 9. Comparison of connectivity in dauer and adult networks using type classification from Witvliet et al[5].

a

Witvliet et al. (2021)	Cook et al. (2019) & Brittin et al. (2021)	This paper	Timepoint	No. of neurons (in NR)	Added neurons
L1-1 (dataset1)			0hr (25°C)	161	
L1-2 (dataset2)			5hr (25°C)	162	L1-1 + CANL
L1-3 (dataset3)			8hr (25°C)	164	L1-2 + SABVL, SABVR
L1-4 (dataset4)			16hr (25°C)	172	L1-3 + ALNL, ALNR, AQR, AVFL, AVFR, AVM, RMHL, RMHR
L2 (dataset5)			23hr (25°C)	174	L1-4 + RMFL, RMFR
L3 (dataset6)			27hr (25°C)	174	
		Dauer	48~72hr (25°C)	181	L3 + HSNL, PLNL, PLNR, PVNL, PVNR, SDQL, SDQR
	L4 (JSH)		L4	180	L3 + HSNL, PLNL, PLNR, PVNL, SDQL, SDQR
Adult-1 (dataset7)			45hr (25°C)	182	L4 + HSNR, PVNR
Adult-2 (dataset8)			45hr (25°C)	182	
	Adult-3 (N2U)		Three-day adult	182	

b

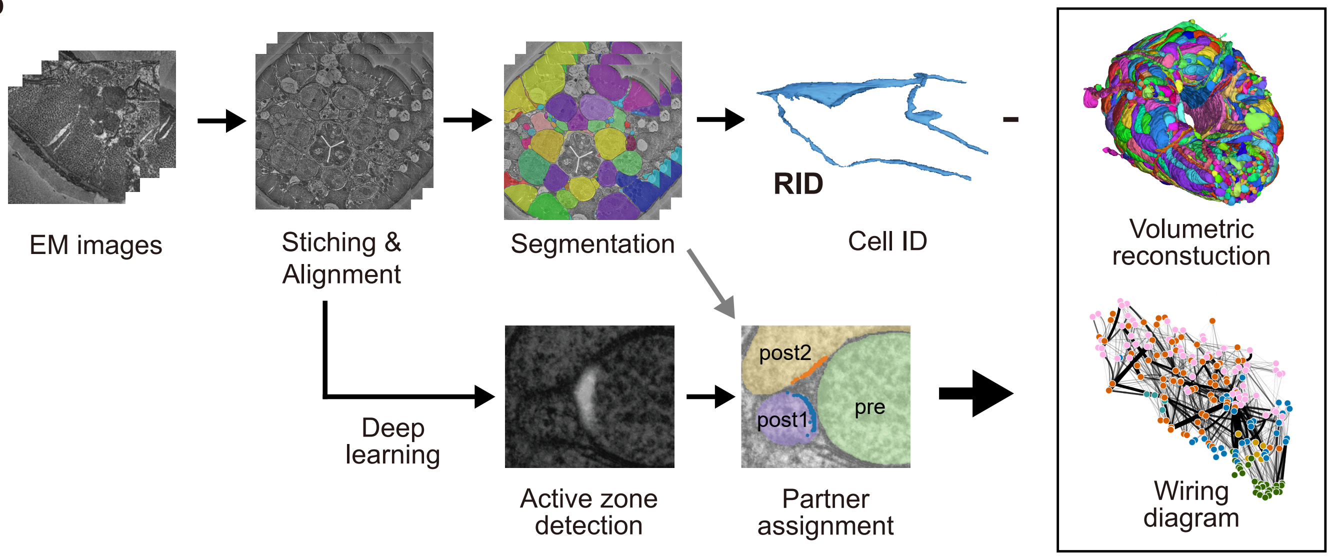

c

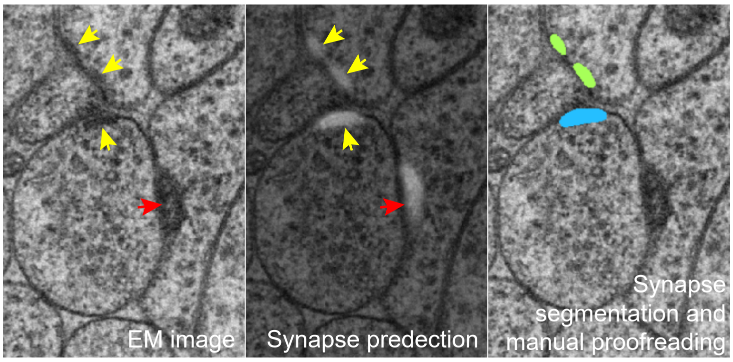

d

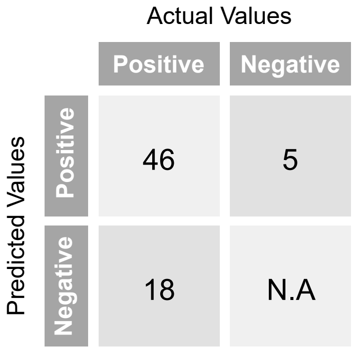

Supplementary Fig. 1. Reconstruction pipeline and comparison with other datasets.

a EM connectome datasets in different developmental stages used for comparison. The number of neurons present in the nerve ring increases as the worm matures. PVNR neuron already exists in the dauer stage while it does not exist until L4 stage and appears in the adult stage. Including CAN neurons.

b Reconstruction pipeline of dauer connectome: alignment of EM images, cell reconstruction and identification, and synapse detection. Electron microscopy (EM) image tiles were stitched and the stitched sections were aligned. From the aligned image stack, annotators manually traced and colored the area inside the membrane for each cell. Classes of reconstructed cells were identified according to morphological features. Chemical synapses were identified by detecting active zones in the EM images using convolutional neural network (CNN). For each active zone, the synaptic partners were assigned, giving a complete connectivity graph of reconstructed neurons.

c Synapse detection in sample EM image cutout. Probability of pixels being in the darkly stained presynaptic active zones (yellow arrow) in the EM image (left) are predicted using CNN (middle, white indicates higher probability). Then for each predicted active zone, the probability values were thresholded and grouped using connected components to segment individual active zones (right, labeled with different colors). Lastly, manual proofreading has been done to remove falsely predicted active zones (red arrow) or falsely assigned active zones.

d Confusion matrix of synapse detection model. The numbers indicate the number of active zones. Since the active zone segments cannot be defined in unpredicted regions, true negatives cannot be quantified.

Neuron	Representative morphological changes in dauer	Schematic view of the changes in dauer (red)
ALA	(B) Branches toward to posterior, receive inputs mainly from ASK neurons	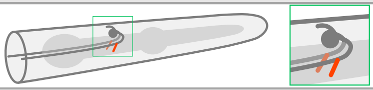
AVB	(P) Protrusions, receive inputs mainly from RIC neurons	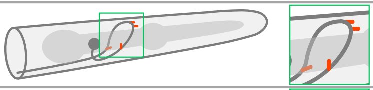
CEP (dorsal)	(P) Upper processes contact together, making dauer-specific gap junction with each other	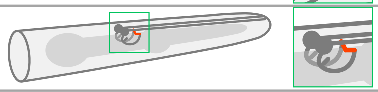
IL2 (quadrant)	(P) Protrusions at the axon bend, dorsal protrusions give output mainly to SAA neurons	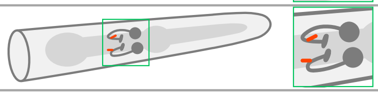
IL2 (lateral)	(B) (S) Branches from axon bend make new connections, terminal swelling shrinks and loses synapses mainly with OLQ	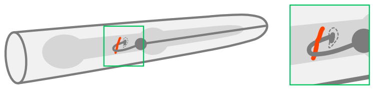
PVC	(R) Two processes stop at the dorsal midline, but no longer overlap	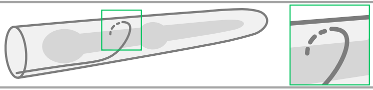
RIH	(B) Small branches at various regions, receive inputs from many neurons (such as DVC)	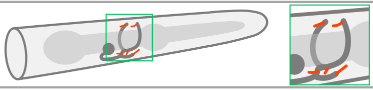
RIR	(R) Processes no longer meet at the dorsal midline	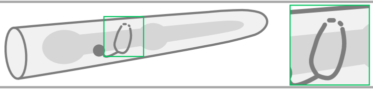
RIV	(R) Branches shortened at ventral midline, lose contacts and synapses with muscle arms	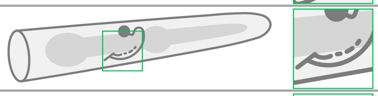
RMD (lateral)	(B) Branches receive inputs from many neurons (such as RME, RMH, URB)	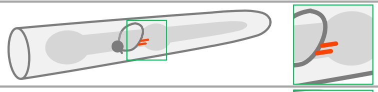
RMF	(B) Branches along lateral processes, receive inputs from many neurons	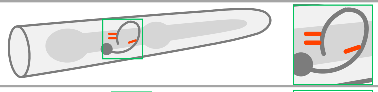
RMG	(P) Protrusions receive inputs mainly from RMD, RMF neurons	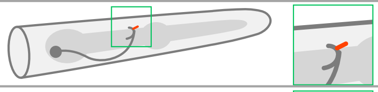
RMH	(B) Branches extend more anteriorly, receive inputs mainly from IL1, RMD neurons	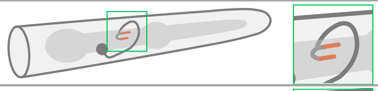
SMD	(B) At the midline, branches extend anteriorly, receive inputs mainly from RME, SMD neurons	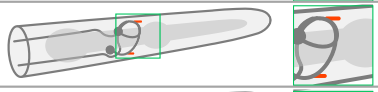
URB	(B) Branches emerge from terminal swelling, receive inputs mainly from IL1 neurons	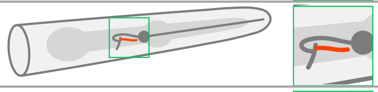
URY	(S) Terminal swelling shrinks, loses most output to	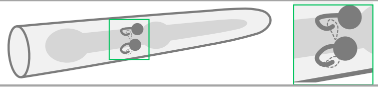

Supplementary Fig. 2 Morphological changes of neurons in the dauer stage.

Summary of representative morphological changes of neurons in the dauer stage with schematic. Morphological changes are classified into 4 types: branching (B), small protrusion (P), retraction (R), and shrinkage (S). Adapted from WormAtlas, Altun, Z.F., Herndon, L.A., Wolkow, C.A., Crocker, C., Lints, R. and Hall, D.H. (ed.s) 2002-2024.

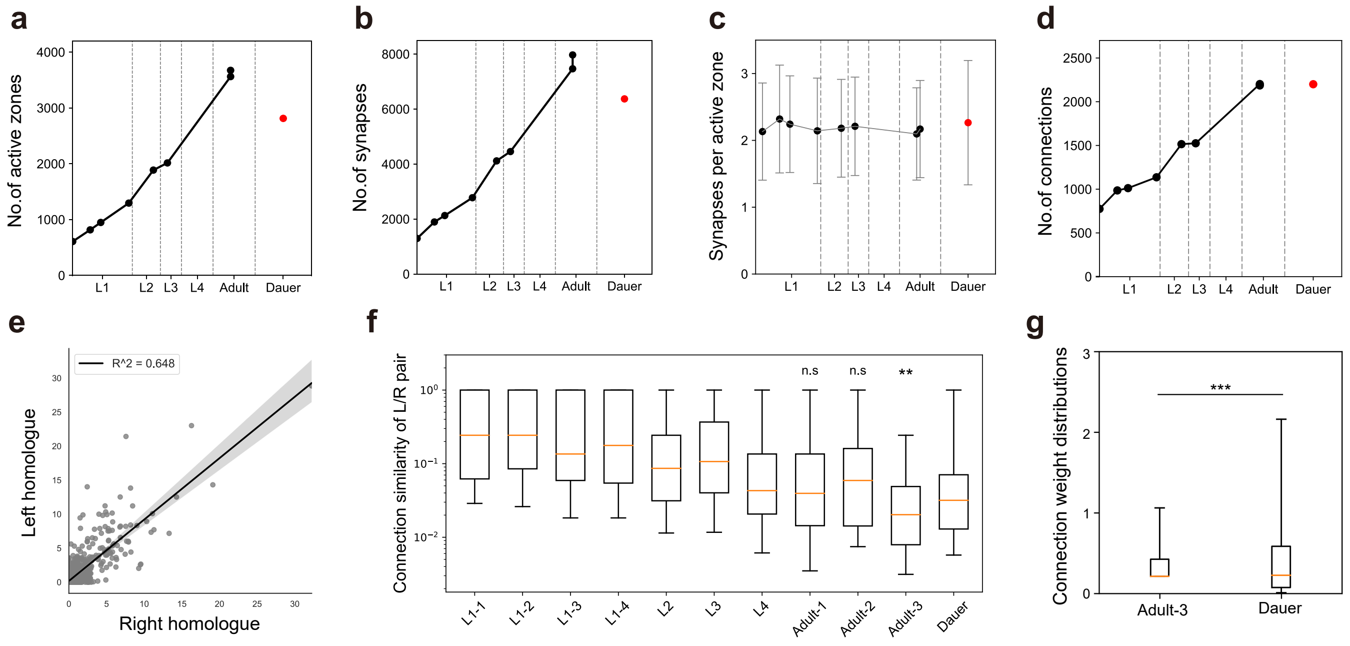

a
No.of active zones
0
1000
2000
3000
4000
L1
L2
L3
L4
Adult
Dauer
b
No.of synapses
0
2000
4000
6000
8000
L1
L2
L3
L4
Adult
Dauer
c
Synapses per active zone
0
1
2
3
L1
L2
L3
L4
Adult
Dauer
d
No.of connections
0
500
1000
1500
2000
2500
L1
L2
L3
L4
Adult
Dauer
e
R^2 = 0.648
Left homologue
Right homologue
0
5
10
15
20
25
30
f
Connection similarity of L/R pair
10^0
10^{-1}
10^{-2}
n.s
n.s
**
L1-1
L1-2
L1-3
L1-4
L2
L3
L4
Adult-1
Adult-2
Adult-3
Dauer
g
Connection weight distributions
***
0
1
2
3
Adult-3
Dauer

Supplementary Fig. 3 Connection properties across development.

a Total number of active zones across development. L4 and adult-3 datasets are excluded as they do not provide synapse information.

b Total number of synapses across development.

c Average number of postsynaptic partners per active zone is consistent across development including the dauer stage (mean±SD).

d Total number of connections including neuromuscular junctions across development.

e Correlations between left and right homologous connections weight(normalized) in dauer. R^2 = 0.648. (Shaded areas around the regression line: 95% confidence intervals. p-value for the regression is 0.0)

f Connection similarity of left/right neuron pairs from each datasets (two-sided Wilcoxon rank-sum test; $^{**}p=8.6\times10^{-3}$). The results showed that the left/right similarity in dauer is not significantly different from, or even better than, that in adults.

g Normalized connection weight distribution of stage-specific connections in dauer dataset and adult-3 dataset (Orange line: median, box: interquartile range, whiskers: 5th and 95th percentiles; two-sided Wilcoxon rank-sum test; $^{***}p=2.2\times10^{-8}$).

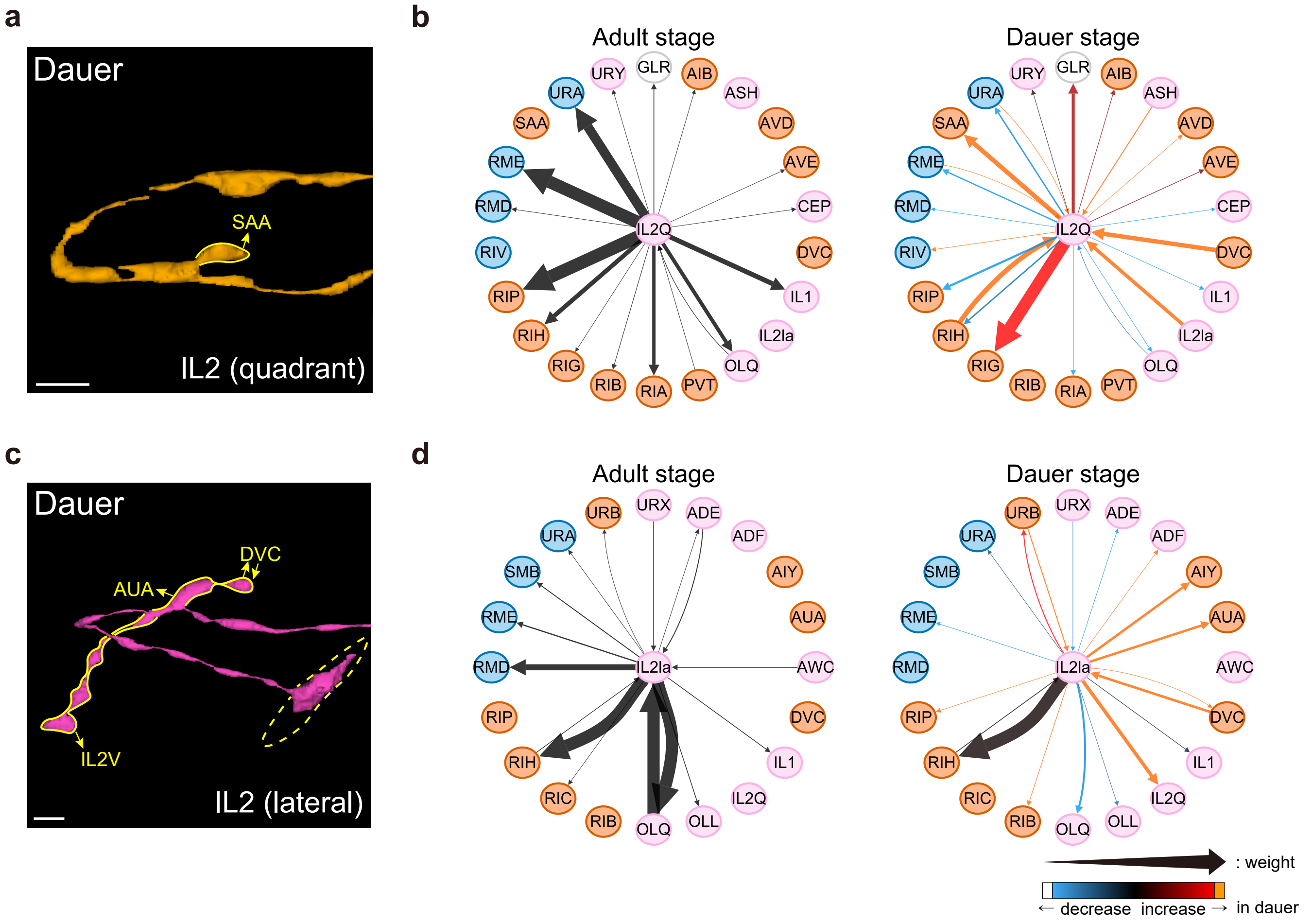

e

Top10 dauer-specifc/increased connection pairs					Top10 dauer loss/decreased connection pairs				
Pair	Connection		Contact		pair	Connection		Contact	
	Adult-2	Dauer	Adult-2	Dauer		Adult-2	Dauer	Adult-2	Dauer
ASG-ASI	0	61.1017	7.8150	17.3504	URY-RMD	26.7482	3.7355	31.1324	12.7418
RIC-AVB	0	46.4916	3.0562	18.2616	IL2-RIP	25.5493	2.0841	19.6557	10.7621
IL1-RIC	0	24.3758	0.7496	4.7401	URY-SMD	23.9327	1.0753	4.8239	3.4658
IL1-RMH	0.5906	20.5621	2.3639	15.2630	ASG-AIA	22.1055	0	11.5422	3.1668
ASI-ASG	1.0014	19.3388	7.8150	17.3504	IL2-RME	19.7697	1.2189	39.8809	24.3689
RME-RMD	0.1965	12.1317	17.5169	21.7359	RIH-RIP	17.4103	2.5771	11.6206	2.0065
IL1-URB	0	11.9600	4.0512	16.6687	URB-URX	17.2857	0	6.5142	1.1784
IL2-RIG	0.0487	10.0102	0.5344	2.9476	IL2-OLQ	15.0634	1.9273	14.0205	16.9633
URB-RMD	0.5609	8.4870	2.0898	6.5734	ALM-BDU	12.5702	1.0948	6.1831	2.5627
ASH-AVJ	0.2166	6.4948	0.6932	6.7006	OLQ-IL2	12.2187	0.2148	14.0205	16.9633

Supplementary Fig. 4 Morphological changes are accompanied by new synaptic inputs and outputs.

a 3D view of an IL2 quadrant neuron (IL2DR) in dauer with new synaptic inputs and outputs (yellow arrow) in the region where morphological changes occurred (solid yellow line).

b Wiring diagram of IL2 quadrant neurons in adult-2 (left) and dauer stages (right). The width and color of the arrow follow the same rule from Figure 2D.

c Same with **a** for an IL2 lateral neuron (IL2R).

d Same with **b** for IL2 lateral neurons.

e Normalized connection weights and contact areas for 10 pairs of neurons with greatest increase in connection weights (left) and greatest decrease in connection weights (right) in dauer relative to adult-2 dataset.

a, c Scale bars: 1 µm.

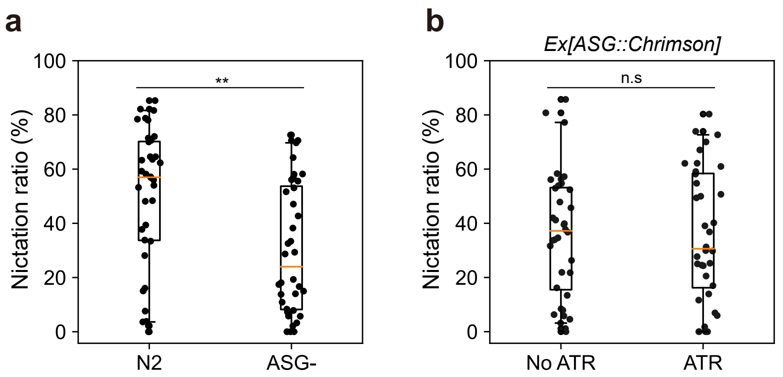
a
b
Ex[ASG::Chrimson]
Nictation ratio (%)
100
80
60
40
20
0
**
n.s
N2
ASG-
No ATR
ATR

Supplementary Fig. 5. Connectivity changes in dauer neurons are correlated with unique behavior in dauer.

a ASG ablated lines show significantly lower nictation ratio than N2 (individual nictation test; n=36; two-sided unpaired t-test; $^{**}p=1.2\times10^{-3}$).

b ASG-activated N2 does not show statistically significant nictation ratio (individual nictation test; n=36; two-sided unpaired t-test; p=0.98).

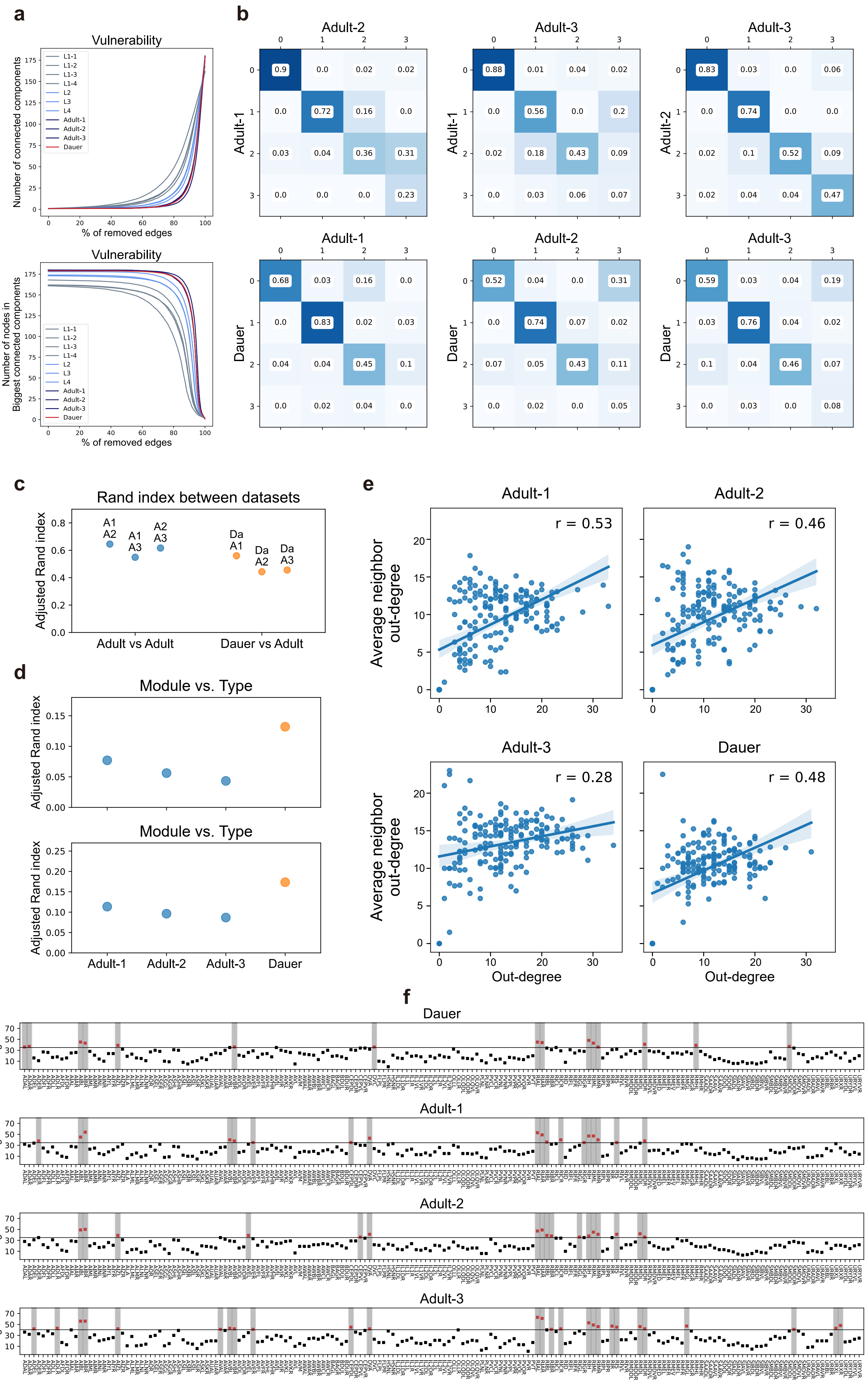
a
Vulnerability
Number of connected components
% of removed edges
L1-1
L1-2
L1-3
L1-4
L2
L3
L4
Adult-1
Adult-2
Adult-3
Dauer
Vulnerability
Number of nodes in
Biggest connected components
% of removed edges
b
Adult-2
Adult-1
Adult-3
Adult-1
Adult-3
Adult-2
Adult-1
Dauer
Adult-2
Dauer
Adult-3
Dauer
c
Rand index between datasets
Adjusted Rand index
A1 A2
A1 A3
A2 A3
Da A1
Da A2
Da A3
Adult vs Adult
Dauer vs Adult
d
Module vs. Type
Adjusted Rand index
Module vs. Type
Adjusted Rand index
Adult-1
Adult-2
Adult-3
Dauer
e
Adult-1
r = 0.53
Adult-2
r = 0.46
Adult-3
r = 0.28
Dauer
r = 0.48
Average neighbor out-degree
Out-degree
f
Dauer
Adult-1
Adult-2
Adult-3
Degree

Supplementary Fig. 6. Other basic network properties.

a Vulnerability of chemical synapse networks across development measured by the number of connected components (top) and the number of nodes in the largest connected component (bottom). Lines are color-coded according to the developmental stages.

b Similarity between modules(subset of neurons) among adults (top row) and between dauer and adults (bottom row). Single-neuron modules from the result are excluded. Order of modules are rearranged for visualization purposes so that the pairs with maximum similarity would be positioned on the main diagonal.

c Rand index of modules between adults (blue) and between dauer and adult (orange). The Rand index quantifies the similarity between two partitions, meaning that each matrix in **b** can be condensed into a single value using the Rand index. The annotation above each dot indicates the datasets being compared (A1: Adult-1, A2: Adult-2, A3: Adult-3, Da: Dauer).

d The Rand index between the modular partitioning and the neuronal class (comprising sensory, inter-, and motor neuron) categorization. The neuronal class classifications in [4] (top) and [5] (bottom) were both used as the basis for assessing the congruence of the partitioned outcomes with the class categorization. (Blue: Adults, Orange: Dauer)

e Correlation between out-degree and average neighbor out-degree of adults and dauer (solid line: linear regression fit, shaded area: 95% confidence interval). In the context of network assortativity, this metric shows the tendency of nodes to establish connections with other nodes that exhibit a similar degree. The results presented herein are based on the out-degree of both the source and target nodes. (r = Pearson correlation coefficient).

f Hub neurons in adults and dauer chemical network based on node out-degree. Horizontal black line indicates the threshold degree(Method). Hub neurons are marked in red and shaded.

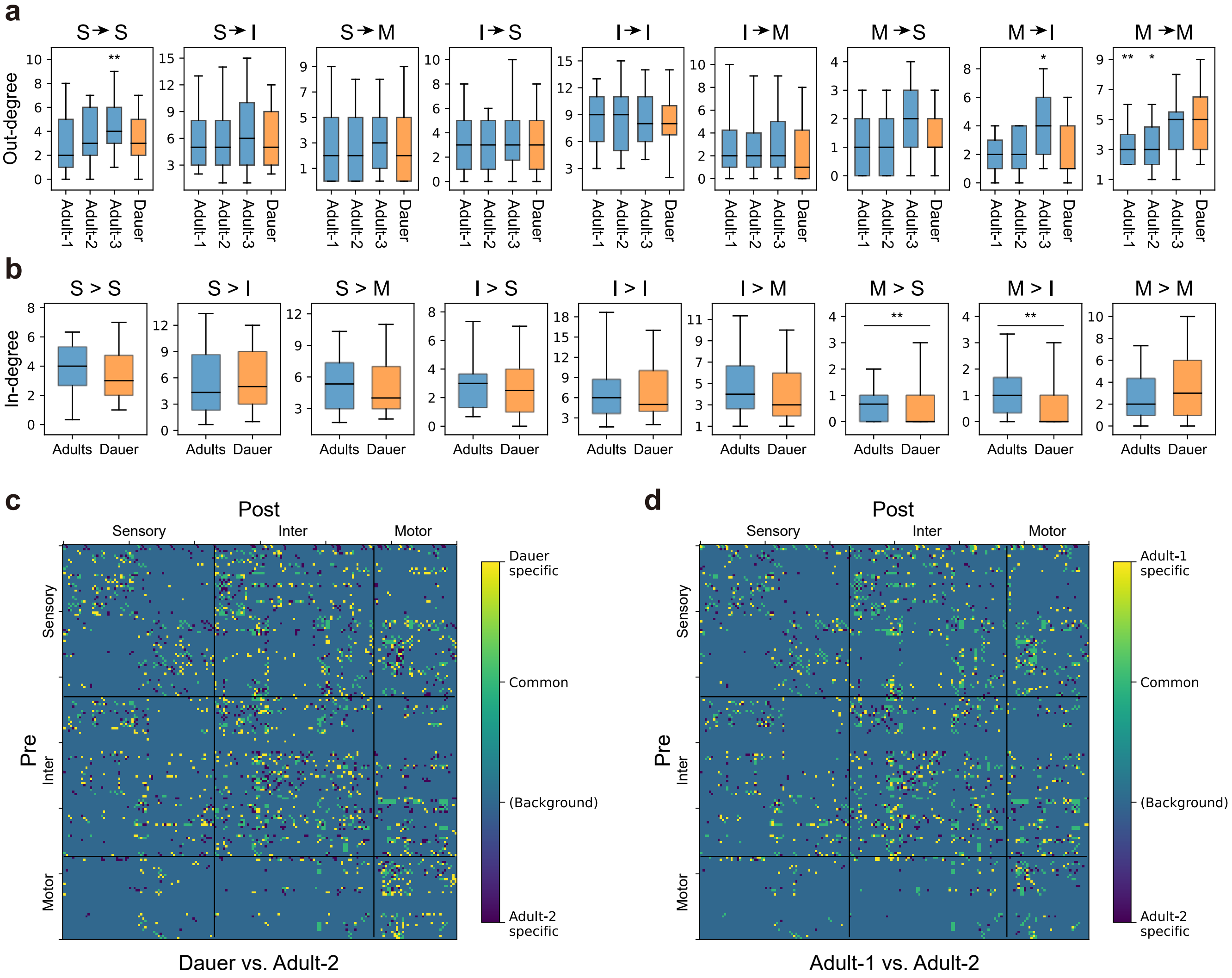
a
S→S
S→I
S→M
I→S
I→I
I→M
M→S
M→I
M→M
Out-degree
Adult-1
Adult-2
Adult-3
Dauer
**
*
b
S > S
S > I
S > M
I > S
I > I
I > M
M > S
M > I
M > M
In-degree
Adults
Dauer
c
Post
Sensory
Inter
Motor
Pre
Dauer specific
Common
(Background)
Adult-2 specific
Dauer vs. Adult-2
d
Adult-1 specific
Adult-1 vs. Adult-2

Supplementary Fig. 7. Type-wise results on out- and in-degree, and whole connectome-wise comparison of dauer and adults.

a Expanded version of Figure 6A (*n*=180; two-sided Wilcoxon rank-sum test; **p*=0.014 (M→I), 0.017 (M→M), ***p*=9.7×10^{-3} (S→S), 3.8×10^{-3} (M→M)).

b In-degrees of neurons for different connection types (two-sided Wilcoxon rank-sum test; n=66(M→S), n=73(M→I), ***p*=3.5×10^{-3} (M→S), 6.9×10^{-3} (M→I)) in adults (blue) and dauer (orange). Neurons without any connection in any of the datasets were excluded. S: sensory, I : inter, M : motor neurons.

c, d Connectivity difference matrices between dauer and adult-2 (**b**) and that between adult-1 and adult-2 (**c**). Connections that are shared between datasets are marked as green and stage-specific connections are marked as yellow or dark blue. Dauer-specific connections (yellow in **b**) are dominant in the motor subnetwork (lower right corner).

a,b Black line: median, box: interquartile range, whiskers: 5th and 95th percentiles.

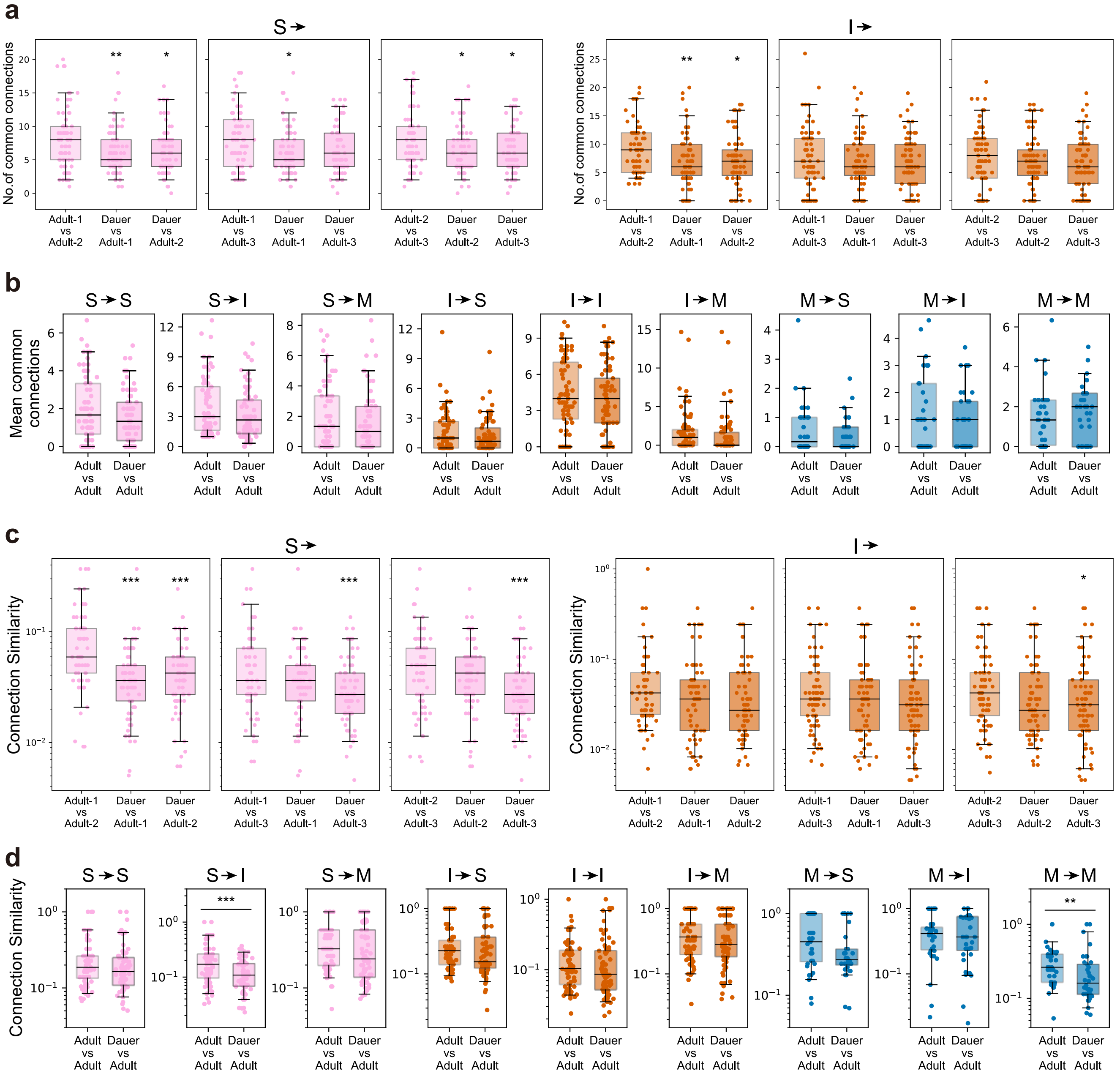
a
S→
I→
No.of common connections
Adult-1 vs Adult-2
Dauer vs Adult-1
Dauer vs Adult-2
Adult-1 vs Adult-3
Dauer vs Adult-3
Adult-2 vs Adult-3
Dauer vs Adult-2
b
S→S
S→I
S→M
I→S
I→I
I→M
M→S
M→I
M→M
Mean common connections
Adult vs Adult
Dauer vs Adult
c
S→
I→
Connection Similarity
d
S→S
S→I
S→M
I→S
I→I
I→M
M→S
M→I
M→M
Connection Similarity
Adult vs Adult
Dauer vs Adult

Supplementary Fig. 8. Expanded and type-wise version of the number of common connections and connection similarity in dauer and adult networks.

a Expanded version of Figure 6E for sensory (pink) and inter- (orange) neurons (two-sided Wilcoxon rank-sum test; **p*=0.017 (S→; left; Dauer vs Adult-2), 0.019 (S→; middle; Dauer vs Adult-1), 0.035 (S→; right; Dauer vs Adult-2), 0.038 (S→; right; Dauer vs Adult-3), 0.014 (I→; left; Dauer vs Adult-2), ***p*=5.4×10^{-3} (S→; left; Dauer vs Adult-1), 9.9×10^{-3} (I→; left; Dauer vs Adult-1)).

b Number of common output connections for different connection types between adults (left) and that between dauer and adults (right). Neurons without any connection in both datasets of the pair were excluded. Colors are based on the presynaptic neuronal class.

c Expanded version of Figure 6F for sensory (pink) and inter- (orange) neurons (two-sided Wilcoxon rank-sum test; **p*=0.047 (I→; right; Dauer vs Adult-3), ****p*=6.1×10^{-7} (S→; left; Dauer vs Adult-1), 2.1×10^{-5} (S→; left; Dauer vs Adult-2), 2.1×10^{-5} (S→; middle; Dauer vs Adult-3), 3.3×10^{-4} (S→; right; Dauer vs Adult-3)).

d Connection similarity of output connections for different connection types between adults (left) and that between dauer and adults (right; two-sided Wilcoxon rank-sum test; ***p*=5.0×10^{-3} (M→M), ****p*=6.7×10^{-4} (S→I)). Neurons without any connection in both datasets of the pair were excluded. Colors are based on the presynaptic neuronal class.

a-d Black line: median, box: interquartile range, whiskers: 5th and 95th percentiles.

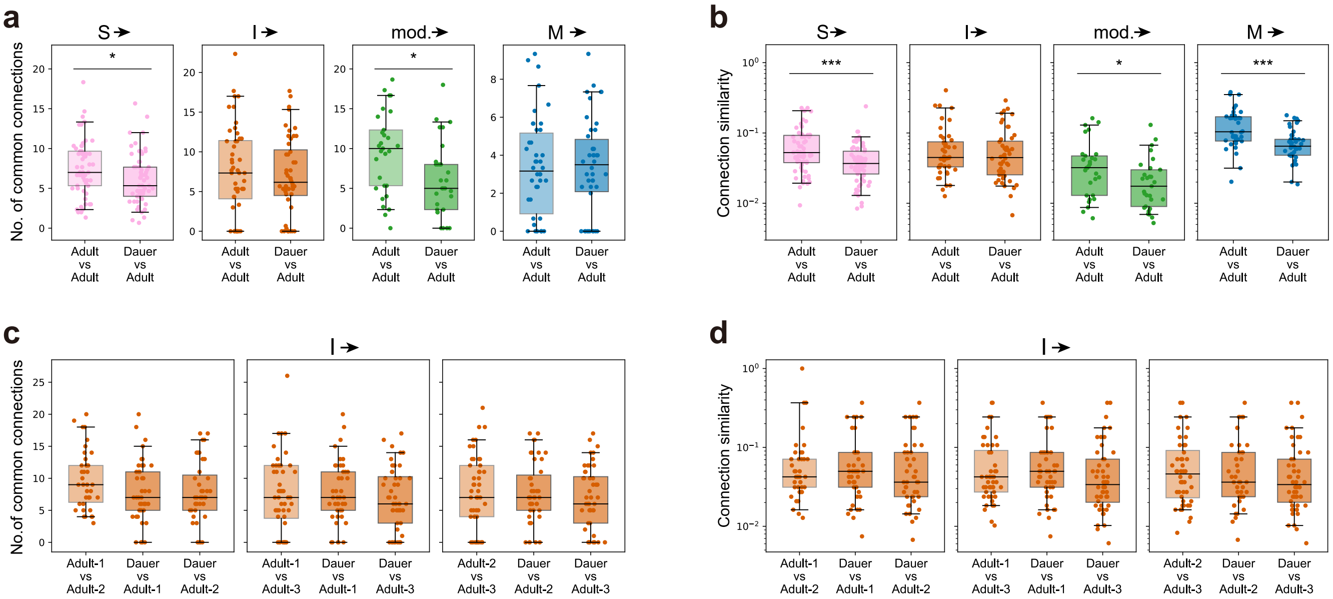
a
b
c
d
S
I
mod.
M
No. of common connections
Connection similarity
No.of common connections
Adult vs Adult
Dauer vs Adult
Adult-1 vs Adult-2
Dauer vs Adult-1
Dauer vs Adult-2
Adult-1 vs Adult-3
Dauer vs Adult-3
Adult-2 vs Adult-3
*
***

Supplementary Fig. 9. Comparison of connectivity in dauer and adult networks using type classification from Witvliet et al[5].

a Number of common output connections from sensory (pink), inter- (orange), modulatory (green), motor (blue) neurons, defined in [5], between adults (left) and that between dauer and adults (right; two-sided Wilcoxon rank-sum test; *p=0.020 (S→), 0.014 (mod.→)).

b Connection similarity of output connections from sensory (pink), inter- (orange), motor (blue) neurons, defined in [5], between adults (left) and that between dauer and adults (right; two-sided Wilcoxon rank-sum test; *p=0.038 (mod.→), ***$p=5.8\times10^{-4}$ (S→), 1.0×10^{-3} (M→)).

c Expanded version of Figure S6A for interneurons defined in [5].

d Expanded version of Figure S6B for interneurons defined in [5].

a-d Black line: median, box: interquartile range, whiskers: 5th and 95th percentiles.
