# Supplementary material for: Comparative connectomics of dauer reveals developmental plasticity
Source: Nat Commun. 2024 Feb 27;15:1546. doi: 10.1038/s41467-024-45943-3 (PMC10899629; doi:10.1038/s41467-024-45943-3)
Supplement: Supplementary file 3 — Description of Additional Supplementary Files [file 41467_2024_45943_MOESM3_ESM.pdf]

Supplementary Data 1. Neuroglancer link and segmentation keys.

Neuroglancer link for the visualization of reconstructed dauer data. 3D reconstructed cells can be visualized with 2D raw EM, cell segmentation, and synapse segmentation images. Segmentation key table includes segment ids of the reconstructed cells in the neuroglancer.

Supplementary Data 2. Dauer synapse list.

Synapses list of dauer connectome with positions and raw connection weights (nm^3).

Supplementary Data 3. Dauer connectivity matrix.

Connectivity matrix of dauer connectome with raw connection weights (nm^3) and normalized connection weights (see Methods). The rows represent presynaptic cells and the columns represent postsynaptic cells. Connectivity matrices of other datasets used in the study are also included as a reference.

Supplementary Data 4. Dauer-specific, dauer-loss, stage-conserved connection lists.

List of dauer-specific, dauer-loss, and stage-conserved connection lists. The normalized weights of each connection in dauer and adult-2 are shown, which have been used to draw comparative wiring diagrams.

Supplementary Data 5. Dauer wiring diagrams.

Wiring diagrams of all neurons. The colors of the nodes represent the cell types (pink: sensory neuron, orange: interneuron, blue: motor neuron, green: body wall muscle). The width of the arrows indicates the relative connection weights. Dauer-specific connections are represented with orange. Connections in which the weight increased and decreased in dauer are represented with red and blue arrows, respectively.

Supplementary Data 6. Dauer contact matrix.

Contact matrix of dauer connectome with raw contact area (nm^2) and normalized contact area (see Methods). The contact matrix is a symmetric matrix as there is no distinction between presynaptic and postsynaptic cells. Contact matrices of other datasets used in the study are also included as a reference.
