# Supplementary material for: Comparative connectomics of dauer reveals developmental plasticity
Source: Nat Commun. 2024 Feb 27;15:1546. doi: 10.1038/s41467-024-45943-3 (PMC10899629; doi:10.1038/s41467-024-45943-3)
Supplement: Supplementary file 8 — Supplementary Data 5 [file 41467_2024_45943_MOESM8_ESM.pdf]

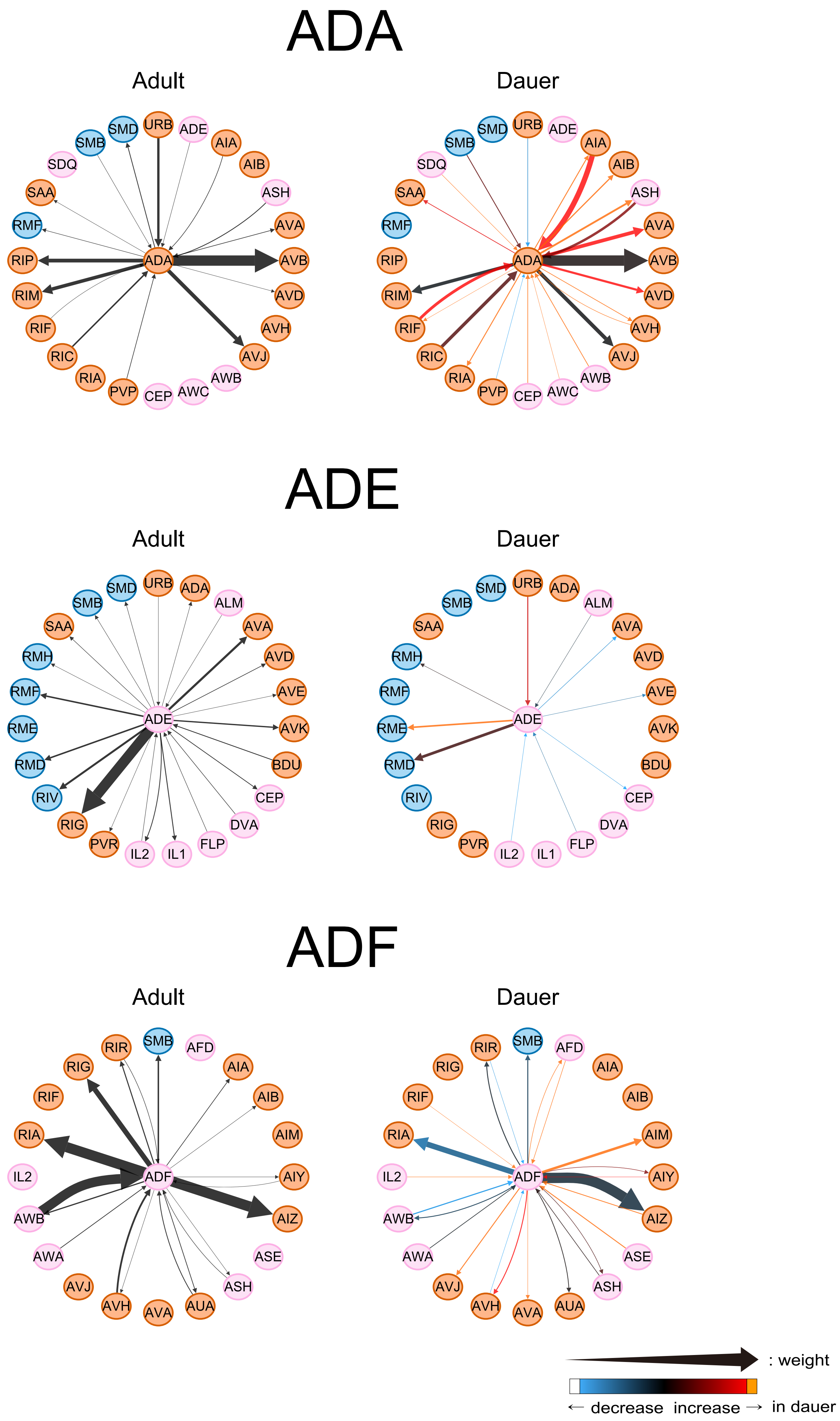
ADA
Adult
Dauer
URB
SMD
SMB
SDQ
SAA
RMF
RIP
RIM
RIF
RIC
RIA
PVP
CEP
AWC
AWB
AVJ
AVH
AVD
AVB
AVA
ASH
AIB
AIA
ADE
ADA
Adult
Dauer
URB
SMD
SMB
SAA
RMH
RMF
RME
RMD
RIV
RIG
PVR
IL2
IL1
FLP
DVA
CEP
BDU
AVK
AVE
AVD
AVA
ALM
ADA
ADE
ADF
Adult
Dauer
SMB
RIR
RIG
RIF
RIA
IL2
AWB
AWA
AVJ
AVH
AVA
AUA
ASH
ASE
AIZ
AIY
AIM
AIB
AIA
AFD
ADF
: weight
← decrease increase → in dauer

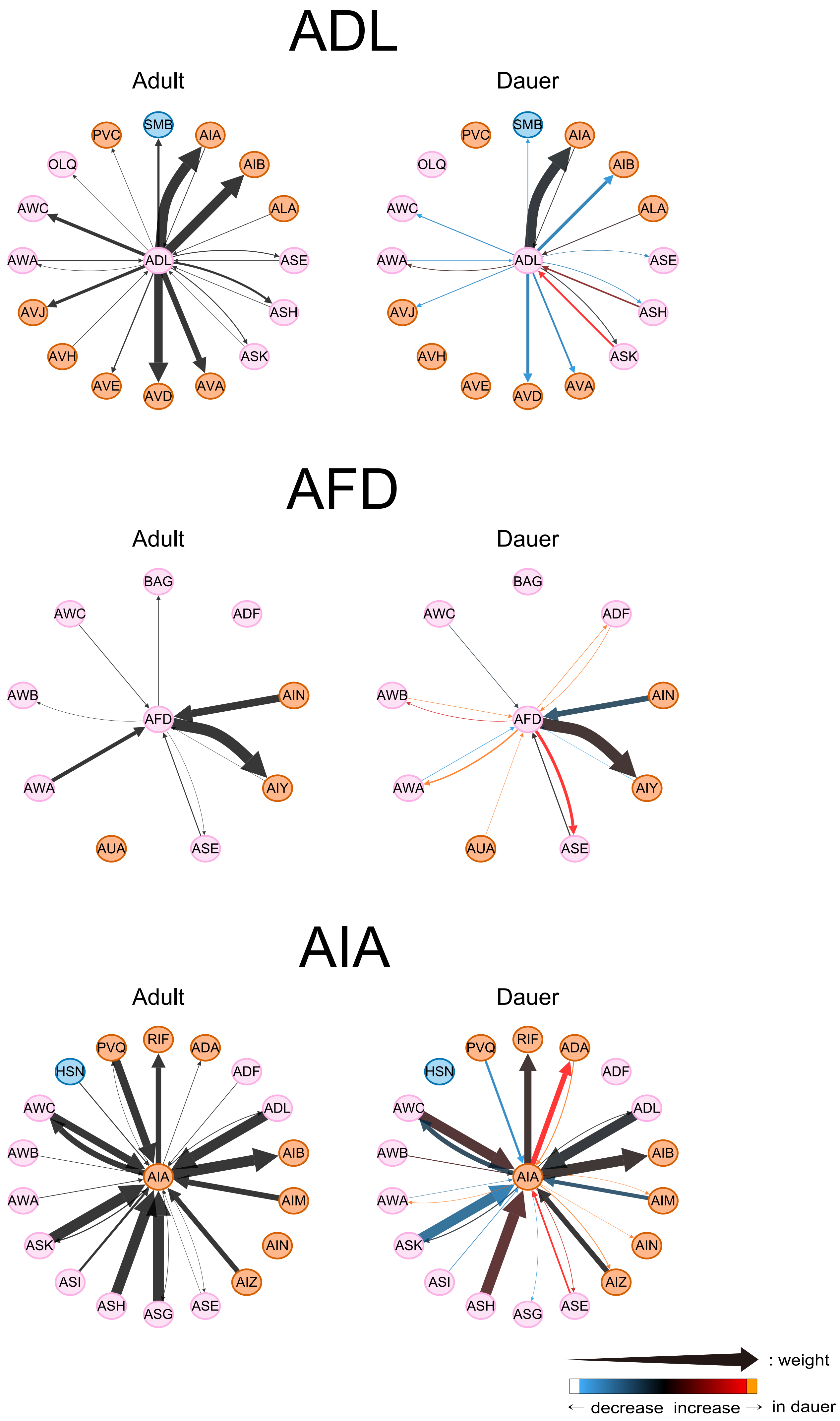
ADL
Adult
SMB
PVC
AIA
OLQ
AIB
AWC
ALA
AWA
ADL
ASE
AVJ
ASH
AVH
ASK
AVE
AVD
AVA
Dauer
SMB
PVC
AIA
OLQ
AIB
AWC
ALA
AWA
ADL
ASE
AVJ
ASH
AVH
ASK
AVE
AVD
AVA
AFD
Adult
BAG
AWC
ADF
AWB
AIN
AFD
AWA
AIY
AUA
ASE
Dauer
BAG
AWC
ADF
AWB
AIN
AFD
AWA
AIY
AUA
ASE
AIA
Adult
RIF
PVQ
ADA
HSN
ADF
AWC
ADL
AWB
AIB
AIA
AWA
AIM
ASK
AIN
ASI
AIZ
ASH
ASG
ASE
Dauer
RIF
PVQ
ADA
HSN
ADF
AWC
ADL
AWB
AIB
AIA
AWA
AIM
ASK
AIN
ASI
AIZ
ASH
ASG
ASE
: weight
← decrease increase → in dauer

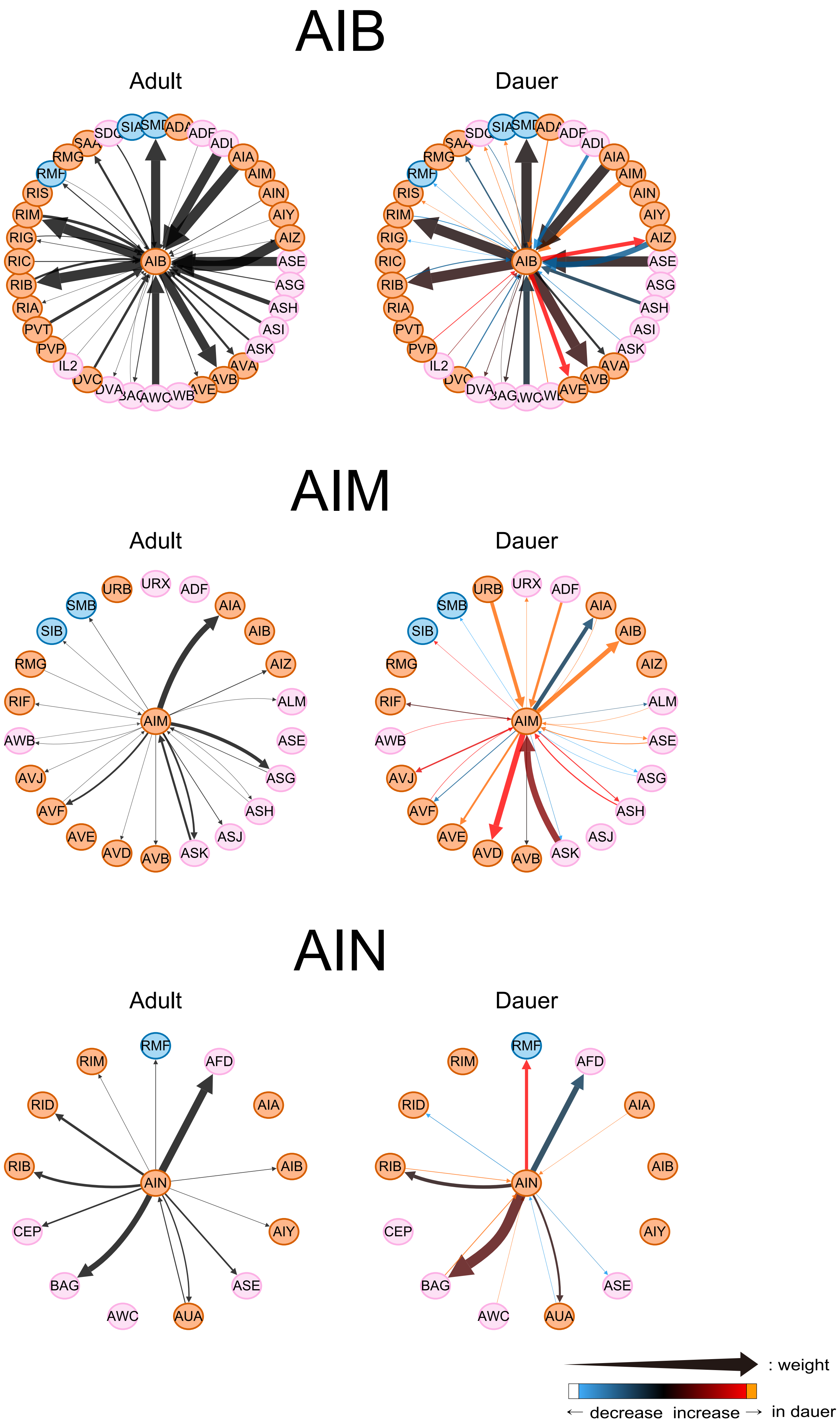
AIB
Adult
Dauer
AIM
Adult
Dauer
AIN
Adult
Dauer
AIB
AIM
AIN
RMG
RIS
RIM
RIG
RIC
RIB
RIA
PVT
PVP
IL2
DVA
AIA
AIY
AIZ
ASE
ASG
ASH
ASI
ASK
AVA
AVB
AVE
URB
URX
ADF
SMB
SIB
RIF
AWB
AVJ
AVF
AVD
ASJ
ALM
RMF
AFD
RID
CEP
BAG
AWC
AUA
: weight
← decrease increase → in dauer

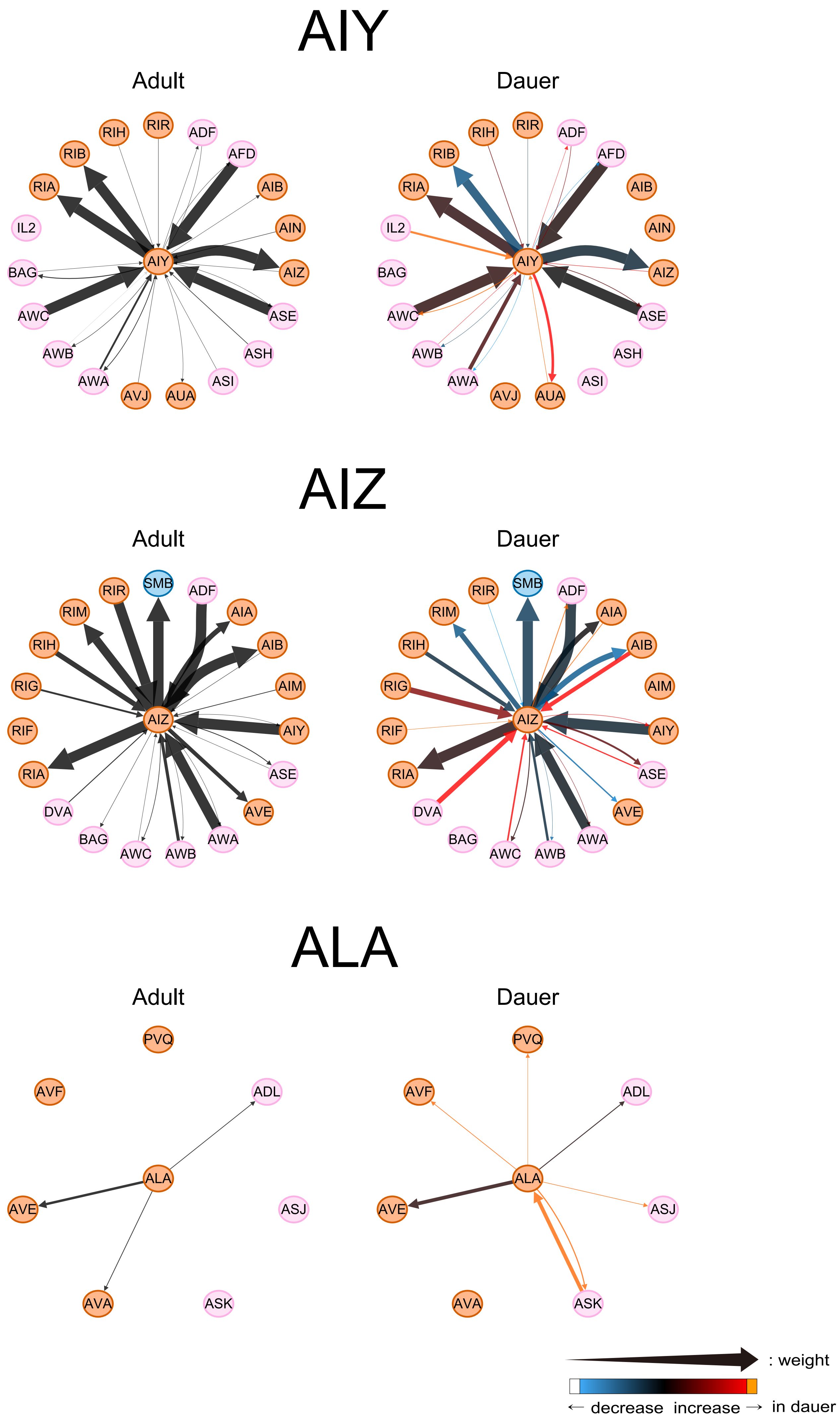
AIY
Adult
Dauer
RIR
RIH
ADF
RIB
AFD
RIA
AIB
IL2
AIN
AIY
BAG
AIZ
AWC
ASE
AWB
ASH
AWA
ASI
AVJ
AUA
AIZ
Adult
Dauer
SMB
RIR
ADF
RIM
AIA
RIH
AIB
RIG
AIM
RIF
AIZ
AIY
RIA
ASE
DVA
AVE
BAG
AWA
AWC
AWB
ALA
Adult
Dauer
PVQ
AVF
ADL
ALA
AVE
ASJ
AVA
ASK
: weight
← decrease increase → in dauer

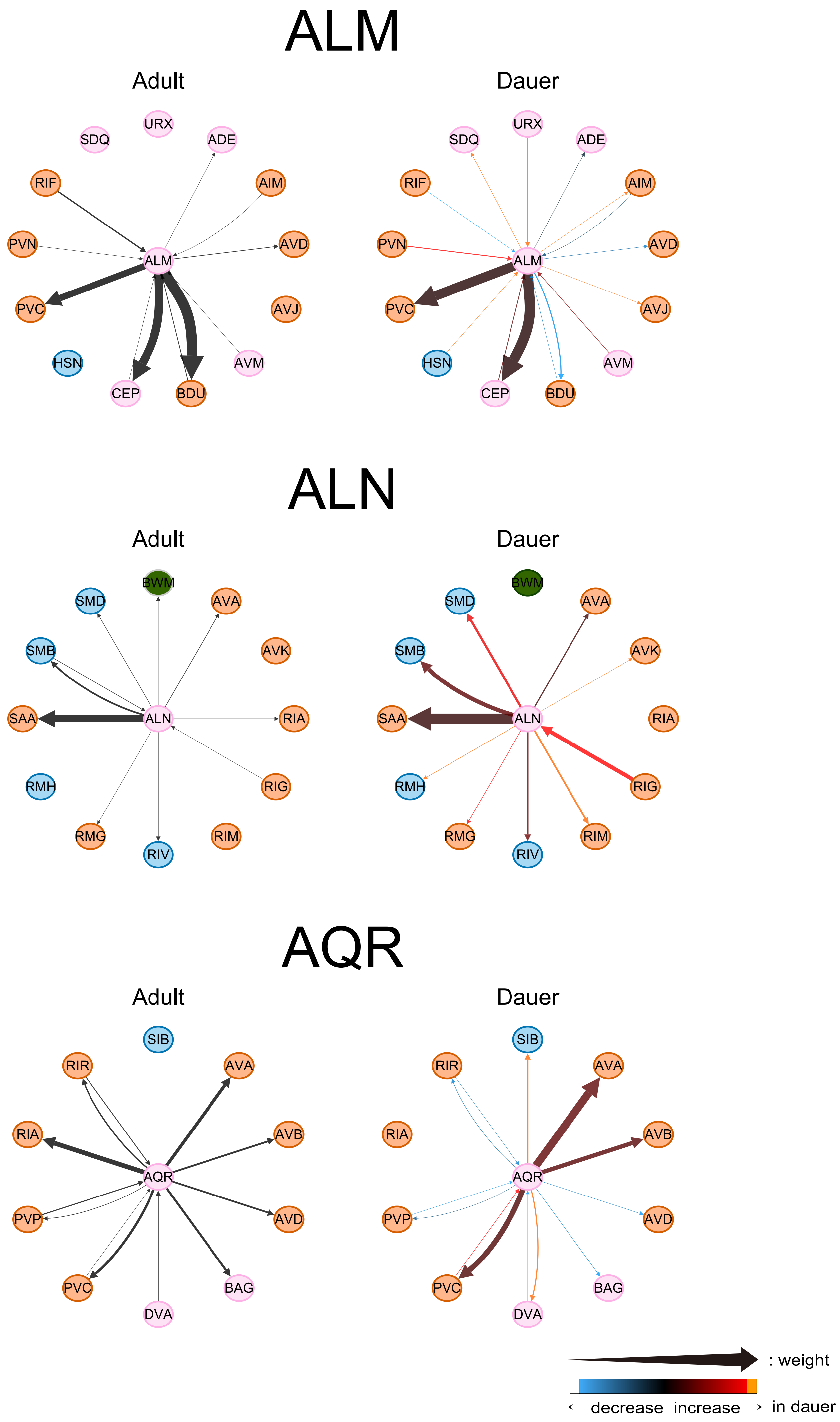
ALM
Adult
Dauer
URX
SDQ
ADE
RIF
AIM
PVN
ALM
AVD
PVC
AVJ
HSN
AVM
CEP
BDU
ALN
Adult
Dauer
BWM
SMD
AVA
SMB
AVK
SAA
ALN
RIA
RMH
RIG
RMG
RIM
RIV
AQR
Adult
Dauer
SIB
RIR
AVA
RIA
AVB
AQR
PVP
AVD
PVC
BAG
DVA
: weight
← decrease increase → in dauer

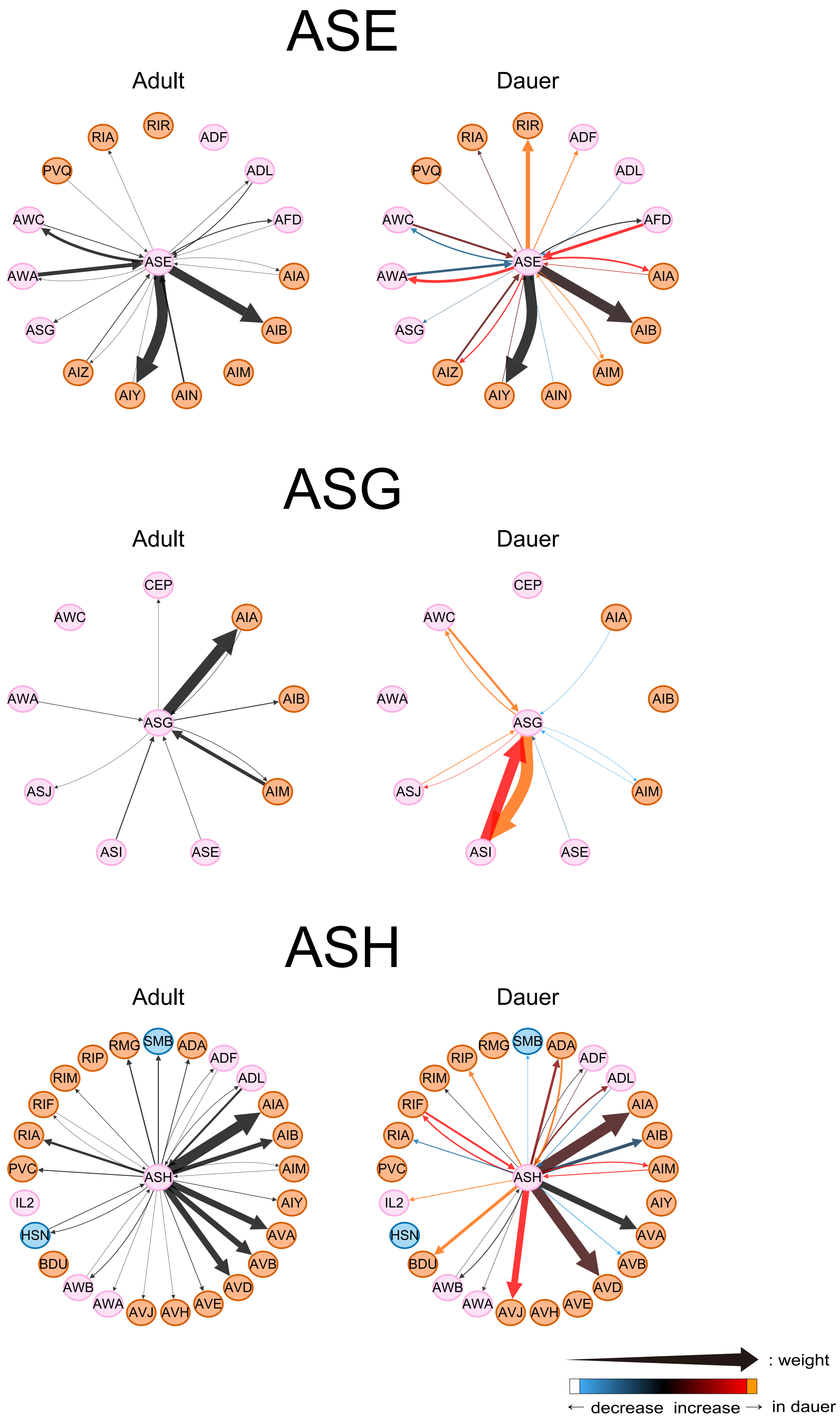
ASE
Adult
Dauer
RIR
RIA
ADF
PVQ
ADL
AWC
AFD
ASE
AWA
AIA
ASG
AIB
AIZ
AIM
AIY
AIN
ASG
Adult
Dauer
CEP
AWC
AIA
AWA
AIB
ASG
ASJ
AIM
ASI
ASE
ASH
Adult
Dauer
RMG
SMB
ADA
RIP
ADF
RIM
ADL
RIF
AIA
RIA
AIB
PVC
ASH
AIM
IL2
AIY
HSN
AVA
BDU
AVB
AWB
AVD
AWA
AVJ
AVH
AVE
: weight
← decrease increase → in dauer

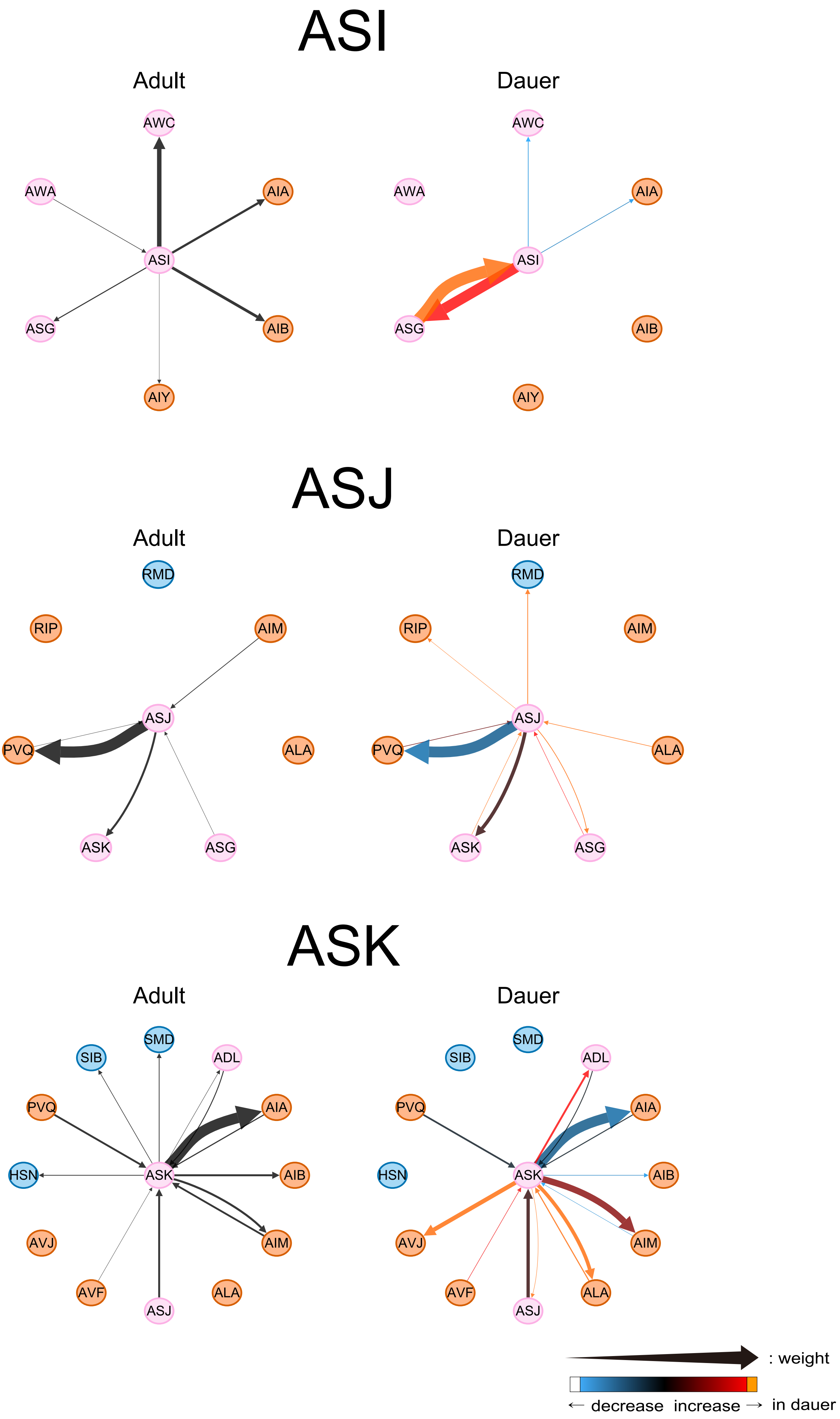
ASI
Adult
Dauer
AWC
AWA
AIA
ASI
ASG
AIB
AIY
ASJ
Adult
Dauer
RMD
RIP
AIM
ASJ
PVQ
ALA
ASK
ASG
ASK
Adult
Dauer
SMD
SIB
ADL
PVQ
AIA
HSN
ASK
AIB
AVJ
AIM
AVF
ALA
ASJ
: weight
← decrease increase → in dauer

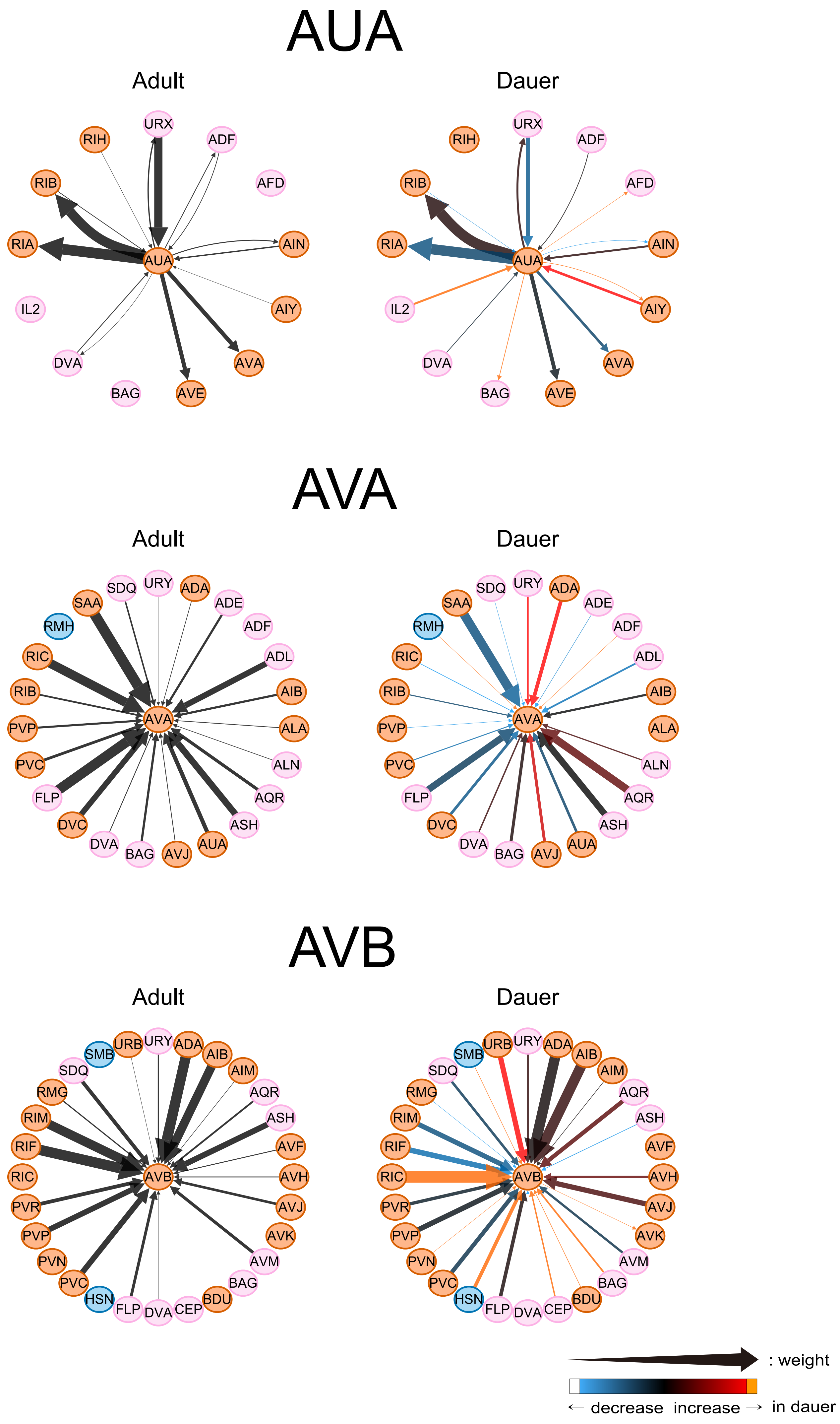
AUA
Adult
Dauer
AVA
Adult
Dauer
AVB
Adult
Dauer
: weight
← decrease increase → in dauer

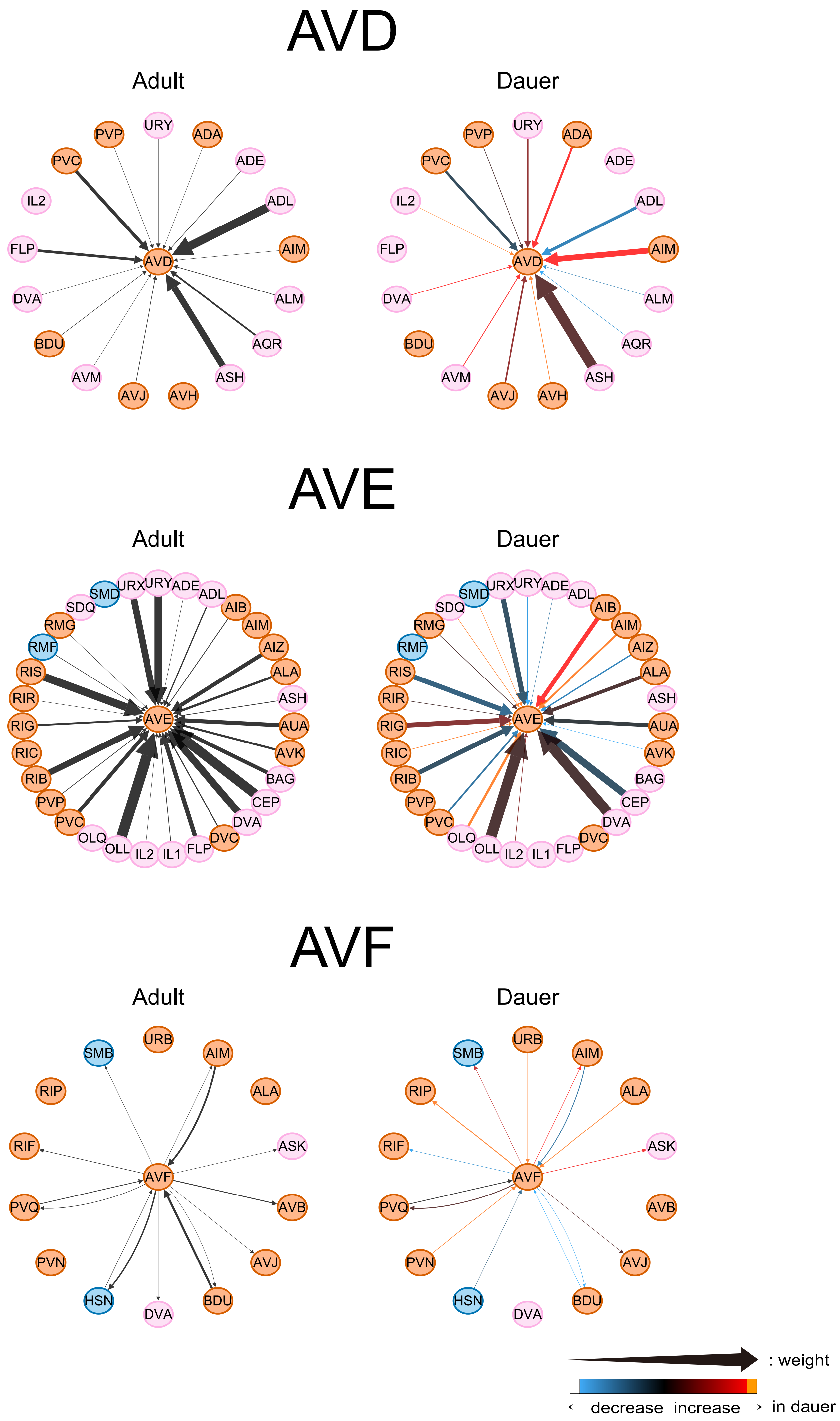
AVD
Adult
Dauer
PVP URY ADA PVC ADE IL2 ADL FLP AIM AVD DVA ALM BDU AQR AVM ASH AVJ AVH
AVE
Adult
Dauer
SMD URX URY ADE ADL SDQ AIB RMG AIM RMF AIZ RIS ALA RIR ASH RIG AVE AUA RIC AVK RIB BAG PVP CEP PVC DVA OLQ DVC OLL IL2 IL1 FLP
AVF
Adult
Dauer
URB SMB AIM RIP ALA RIF ASK AVF PVQ AVB PVN AVJ HSN BDU DVA
: weight
← decrease increase → in dauer

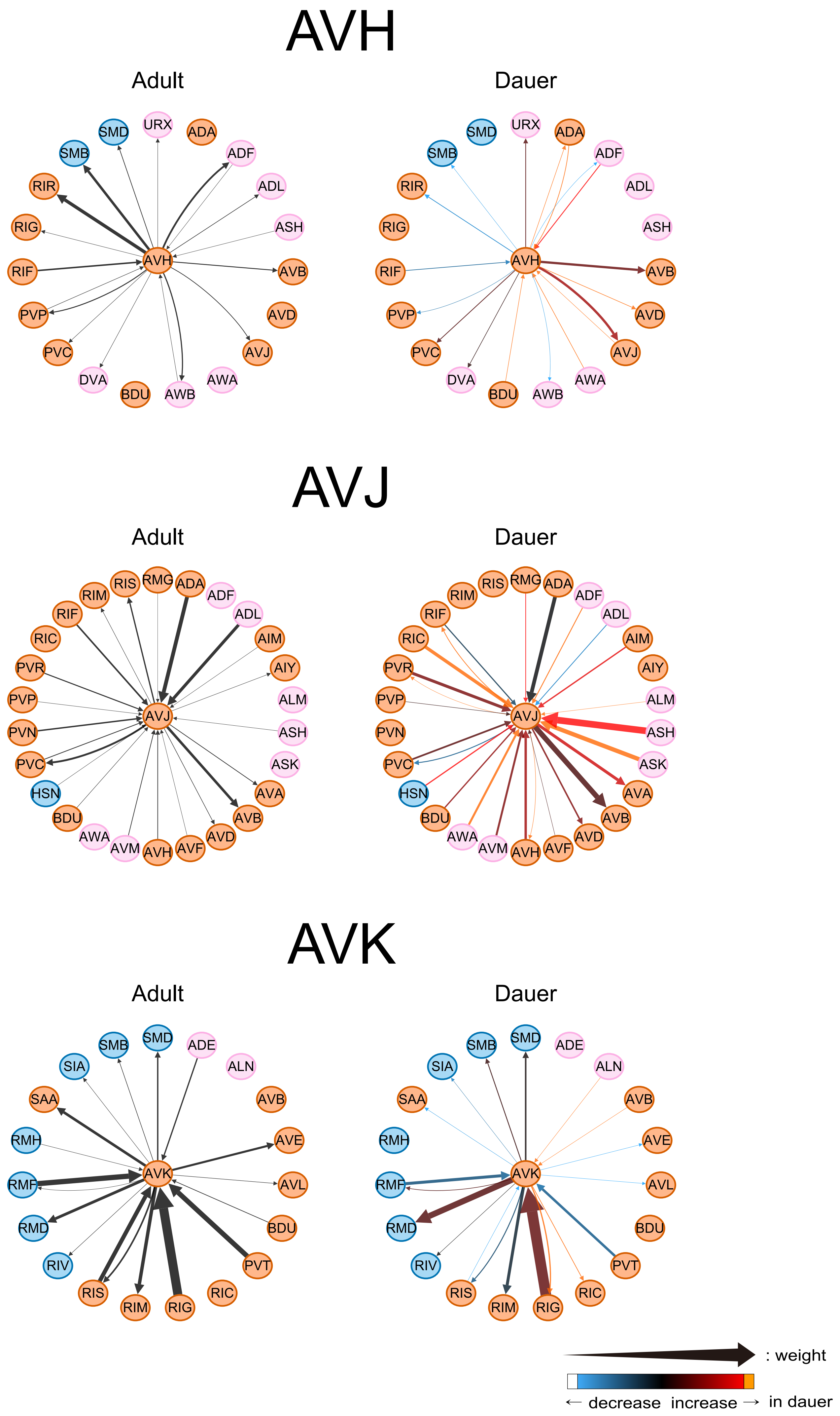

AVH
Adult
Dauer
SMD
URX
ADA
SMB
ADF
RIR
ADL
RIG
ASH
RIF
AVH
AVB
PVP
AVD
PVC
AVJ
DVA
AWA
BDU
AWB
AVJ
Adult
Dauer
RIS
RMG
ADA
RIM
ADF
RIF
ADL
RIC
AIM
PVR
AIY
PVP
ALM
PVN
AVJ
ASH
PVC
ASK
HSN
AVA
BDU
AVB
AWA
AVD
AVM
AVH
AVF
AVK
Adult
Dauer
SMB
SMD
ADE
SIA
ALN
SAA
AVB
RMH
AVE
RMF
AVK
AVL
RMD
BDU
RIV
PVT
RIS
RIC
RIM
RIG
: weight
← decrease increase → in dauer

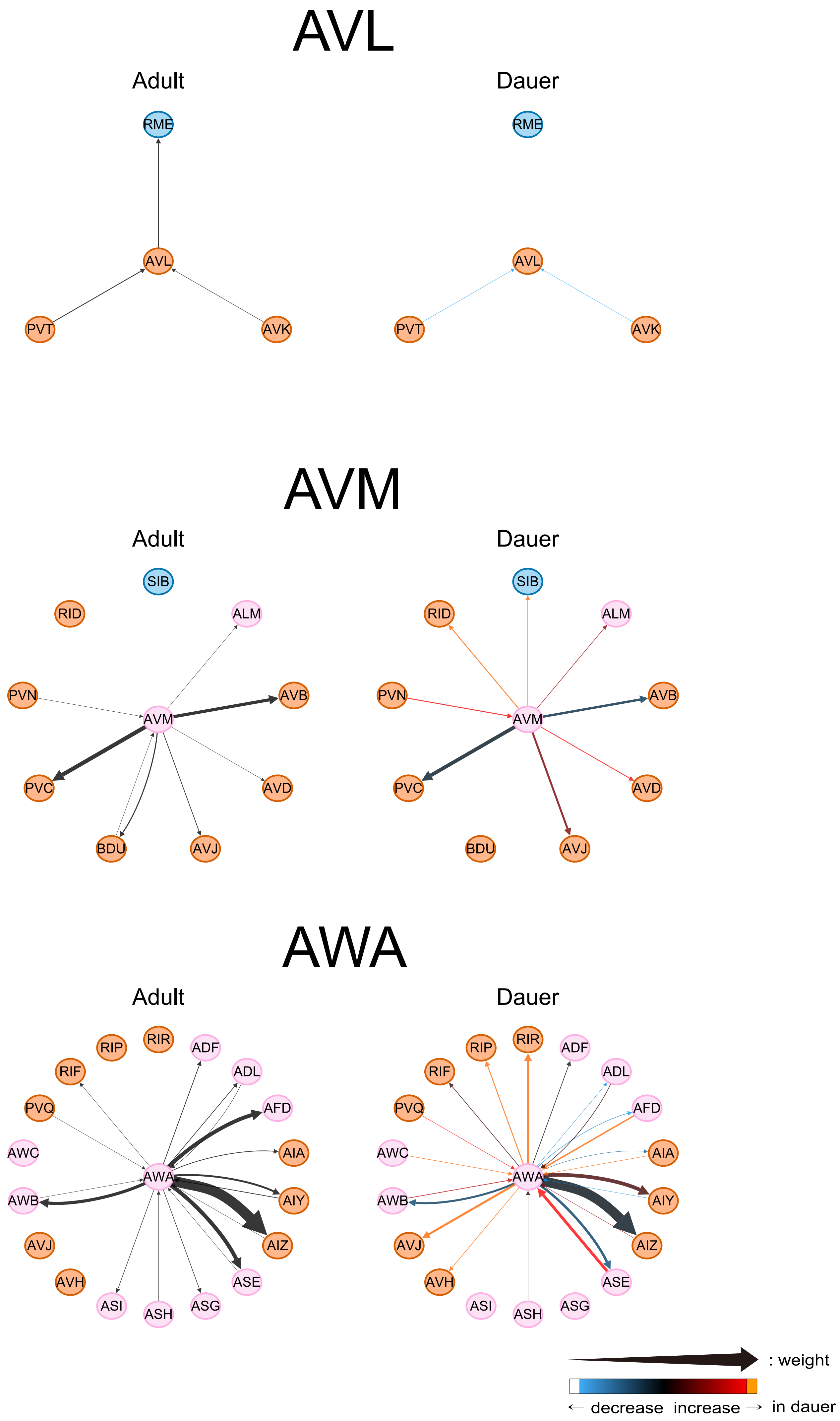

AVL
Adult
RME
AVL
PVT
AVK
Dauer
RME
AVL
PVT
AVK
AVM
Adult
SIB
RID
ALM
PVN
AVB
AVM
PVC
AVD
BDU
AVJ
Dauer
SIB
RID
ALM
PVN
AVB
AVM
PVC
AVD
BDU
AVJ
AWA
Adult
RIP
RIR
ADF
RIF
ADL
PVQ
AFD
AWC
AIA
AWA
AWB
AIY
AVJ
AIZ
AVH
ASE
ASI
ASH
ASG
Dauer
RIP
RIR
ADF
RIF
ADL
PVQ
AFD
AWC
AIA
AWA
AWB
AIY
AVJ
AIZ
AVH
ASE
ASI
ASH
ASG
: weight
← decrease increase → in dauer

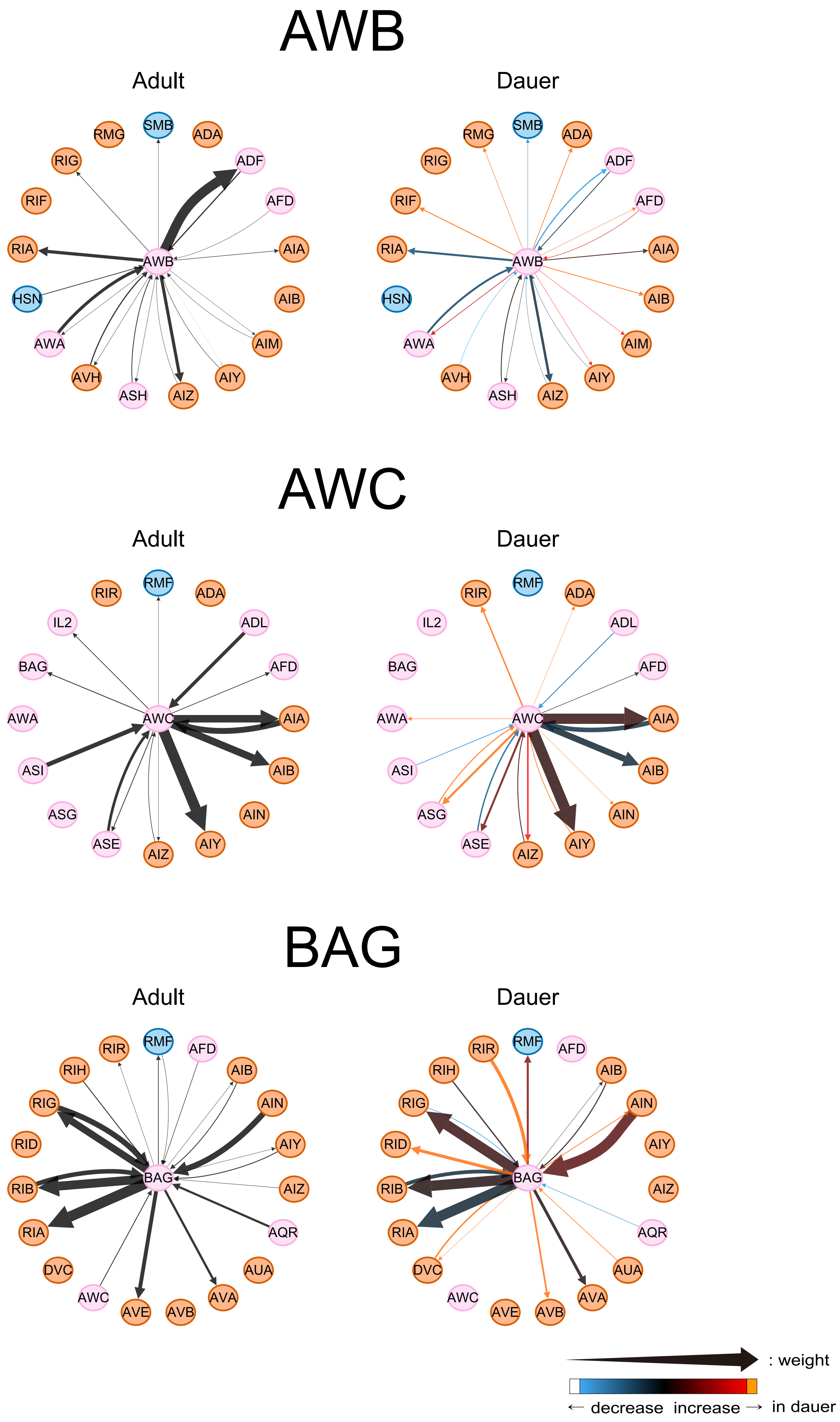
AWB
Adult
Dauer
SMB
RMG
ADA
RIG
ADF
RIF
AFD
RIA
AWB
AIA
HSN
AIB
AWA
AIM
AVH
AIY
ASH
AIZ
AWC
Adult
Dauer
RMF
RIR
ADA
IL2
ADL
BAG
AFD
AWA
AWC
AIA
ASI
AIB
ASG
AIN
ASE
AIY
AIZ
BAG
Adult
Dauer
RMF
RIR
AFD
RIH
AIB
RIG
AIN
RID
AIY
RIB
BAG
AIZ
RIA
AQR
DVC
AUA
AWC
AVA
AVE
AVB
: weight
← decrease increase → in dauer

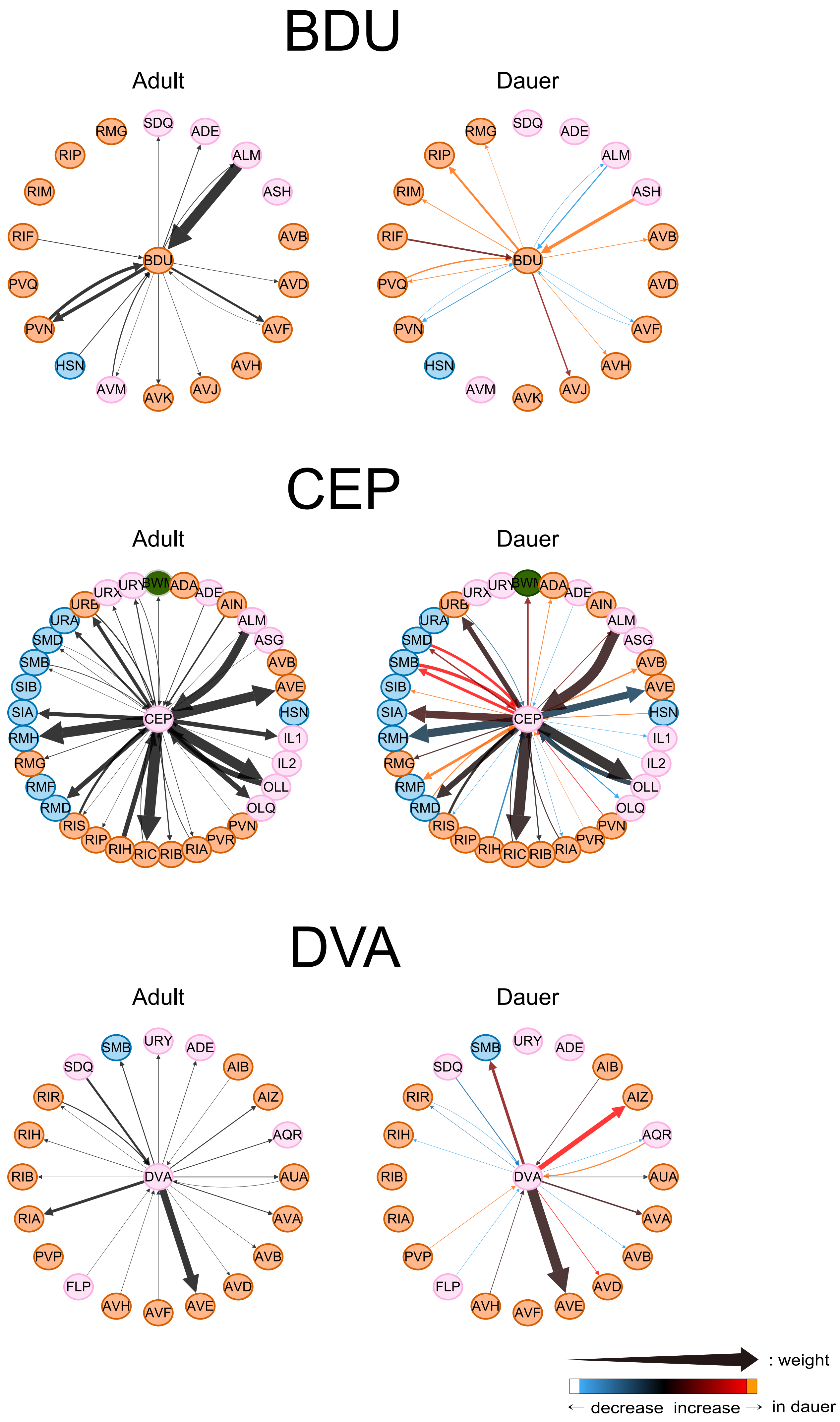

BDU
Adult
Dauer
RMG
SDQ
ADE
RIP
ALM
RIM
ASH
RIF
AVB
PVQ
AVD
PVN
AVF
HSN
AVH
AVM
AVK
AVJ
CEP
URX
ADA
URB
AIN
URA
SMD
ASG
SMB
SIB
AVE
SIA
HSN
RMH
IL1
RMG
IL2
RMF
OLL
RMD
OLQ
RIS
PVN
RIP
PVR
RIH
RIC
RIB
RIA
DVA
SMB
URY
SDQ
AIB
RIR
AIZ
RIH
AQR
RIB
AUA
RIA
AVA
PVP
FLP
AVH
AVF
AVE
AVD
: weight
← decrease increase → in dauer

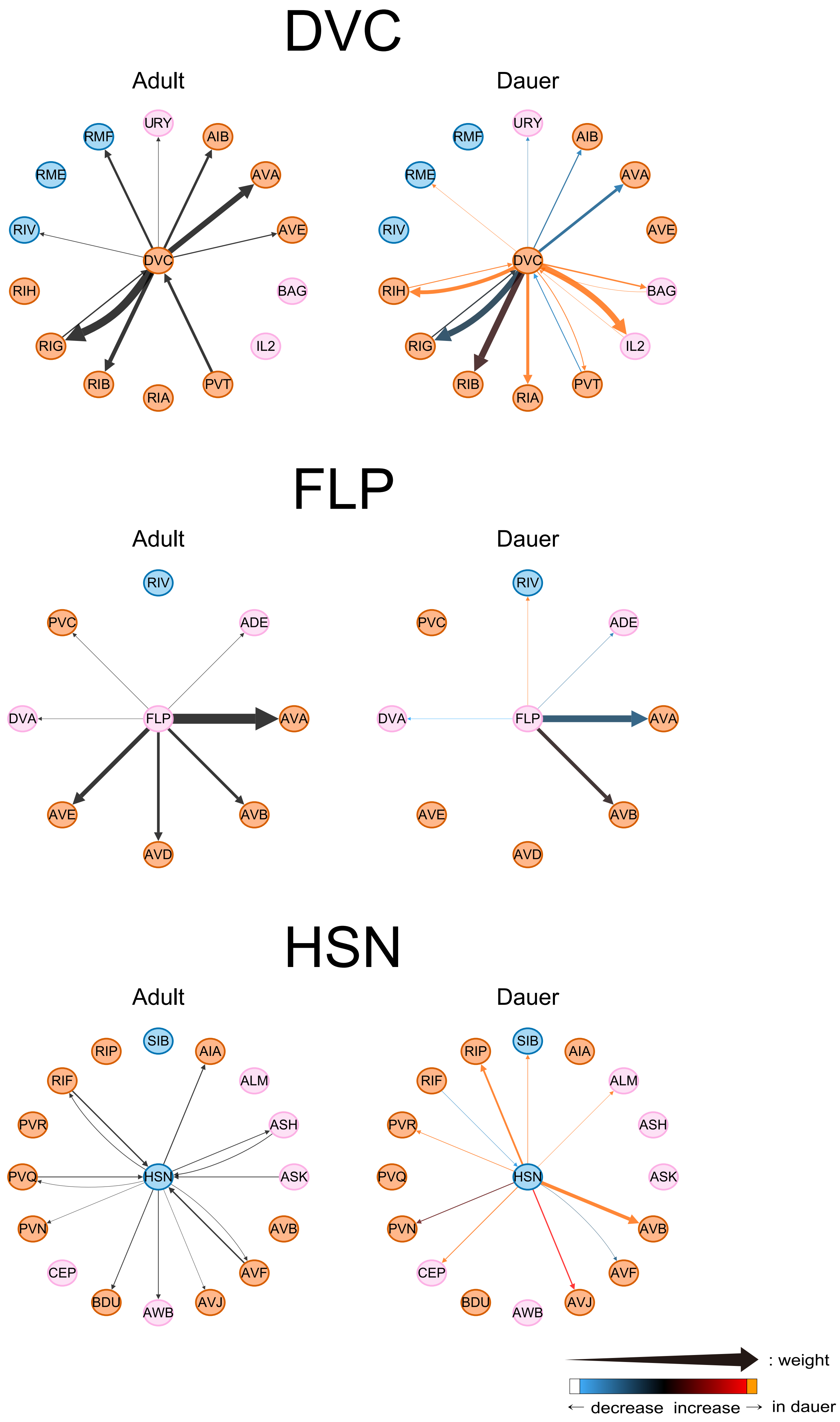
DVC
Adult
Dauer
URY
RMF
AIB
RME
AVA
RIV
AVE
DVC
RIH
BAG
RIG
IL2
RIB
PVT
RIA
FLP
Adult
Dauer
RIV
PVC
ADE
DVA
FLP
AVA
AVE
AVB
AVD
HSN
Adult
Dauer
SIB
RIP
AIA
RIF
ALM
PVR
ASH
PVQ
HSN
ASK
PVN
AVB
CEP
AVF
BDU
AWB
AVJ
: weight
← decrease increase → in dauer

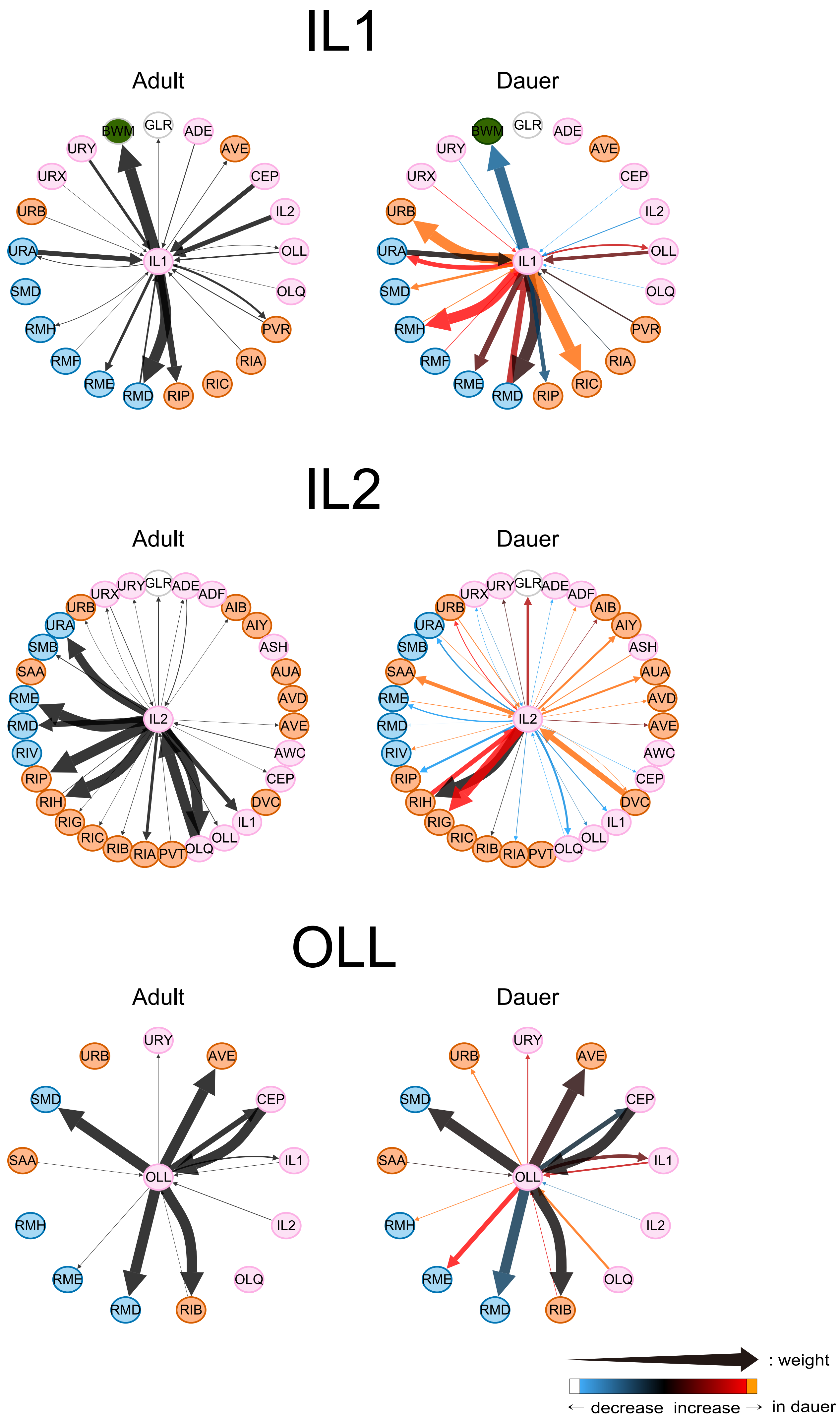

IL1
Adult
Dauer
BWM
GLR
ADE
AVE
CEP
IL2
OLL
OLQ
PVR
RIA
RIC
RIP
RMD
RME
RMF
RMH
SMD
URA
URB
URX
URY
IL2
Adult
Dauer
URX
URY
GLR
ADE
ADF
AIB
AIY
ASH
AUA
AVD
AVE
AWC
CEP
DVC
IL1
OLL
OLQ
PVT
RIA
RIB
RIC
RIG
RIH
RIP
RIV
RMD
RME
SAA
SMB
URA
URB
OLL
Adult
Dauer
URY
URB
AVE
SMD
CEP
SAA
IL1
RMH
IL2
RME
OLQ
RMD
RIB
: weight
← decrease increase → in dauer

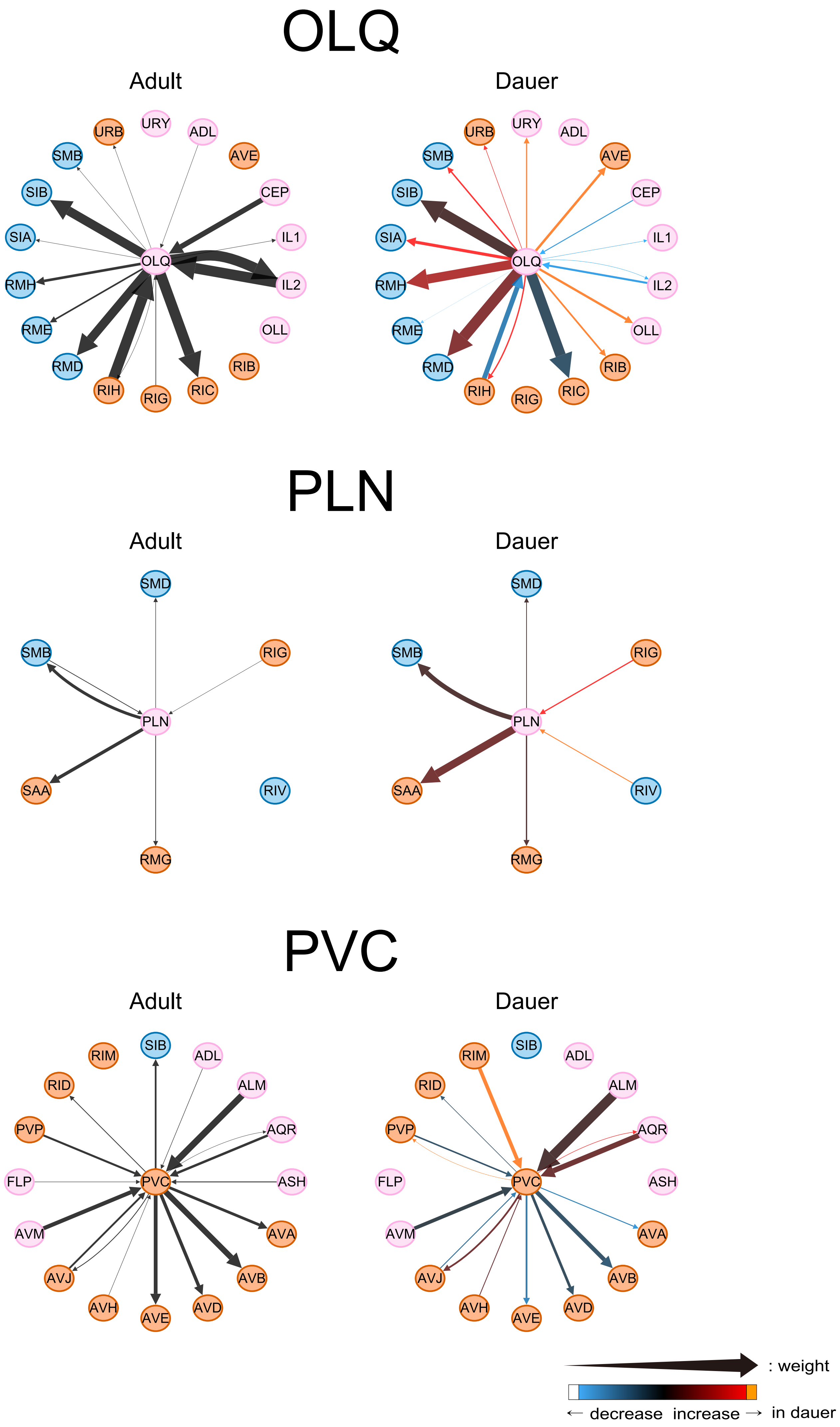

OLQ
Adult
Dauer
URB
URY
ADL
SMB
AVE
SIB
CEP
SIA
IL1
OLQ
RMH
IL2
RME
OLL
RMD
RIB
RIH
RIG
RIC
PLN
Adult
Dauer
SMD
SMB
RIG
PLN
SAA
RIV
RMG
PVC
Adult
Dauer
RIM
SIB
ADL
RID
ALM
PVP
AQR
FLP
PVC
ASH
AVM
AVA
AVJ
AVB
AVH
AVE
AVD
: weight
← decrease increase → in dauer

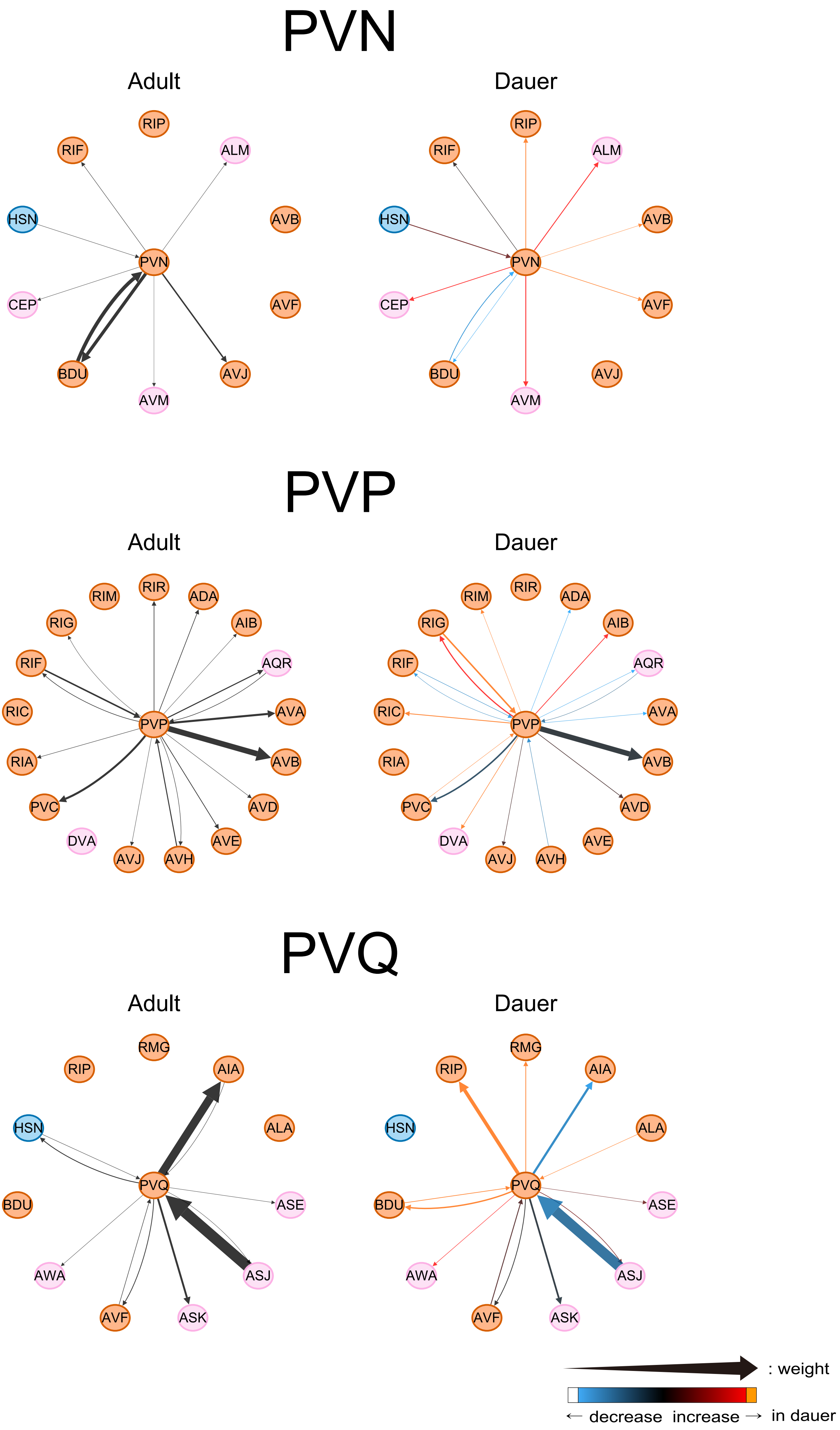
PVN
Adult
Dauer
RIP
RIF
ALM
HSN
AVB
PVN
CEP
AVF
BDU
AVJ
AVM
PVP
Adult
Dauer
RIM
RIR
ADA
RIG
AIB
RIF
AQR
RIC
AVA
PVP
RIA
AVB
PVC
AVD
DVA
AVE
AVJ
AVH
PVQ
Adult
Dauer
RMG
RIP
AIA
HSN
ALA
PVQ
BDU
ASE
AWA
ASJ
AVF
ASK
: weight
← decrease increase → in dauer

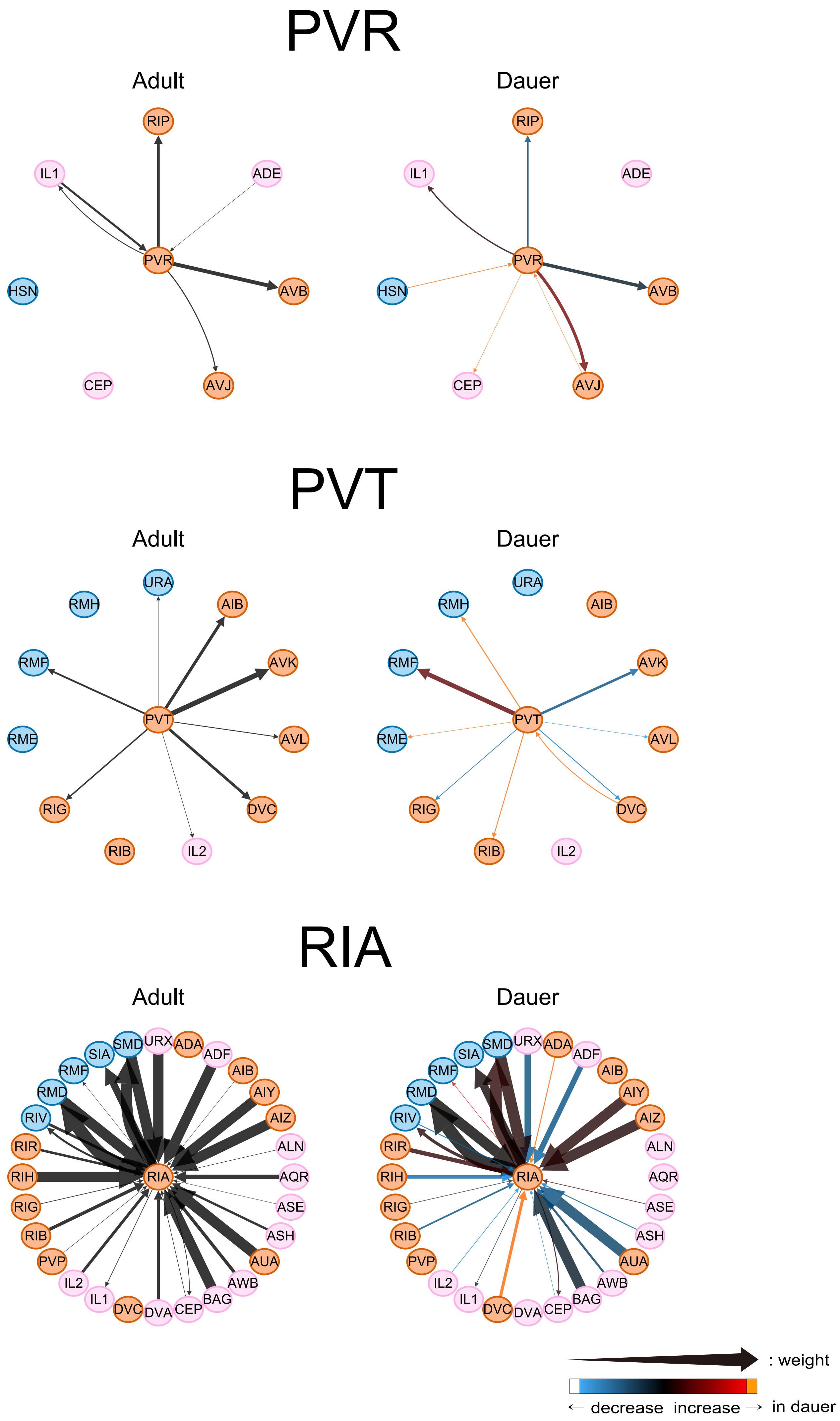
PVR
Adult
Dauer
RIP
IL1
ADE
PVR
HSN
AVB
CEP
AVJ
PVT
Adult
Dauer
URA
RMH
AIB
RMF
AVK
RME
PVT
AVL
RIG
DVC
RIB
IL2
RIA
Adult
Dauer
SMD
URX
ADA
SIA
ADF
RMF
AIB
RMD
AIY
RIV
AIZ
RIR
ALN
RIH
RIA
AQR
RIG
ASE
RIB
ASH
PVP
AUA
IL2
AWB
IL1
BAG
DVC
DVA
CEP
: weight
← decrease increase → in dauer

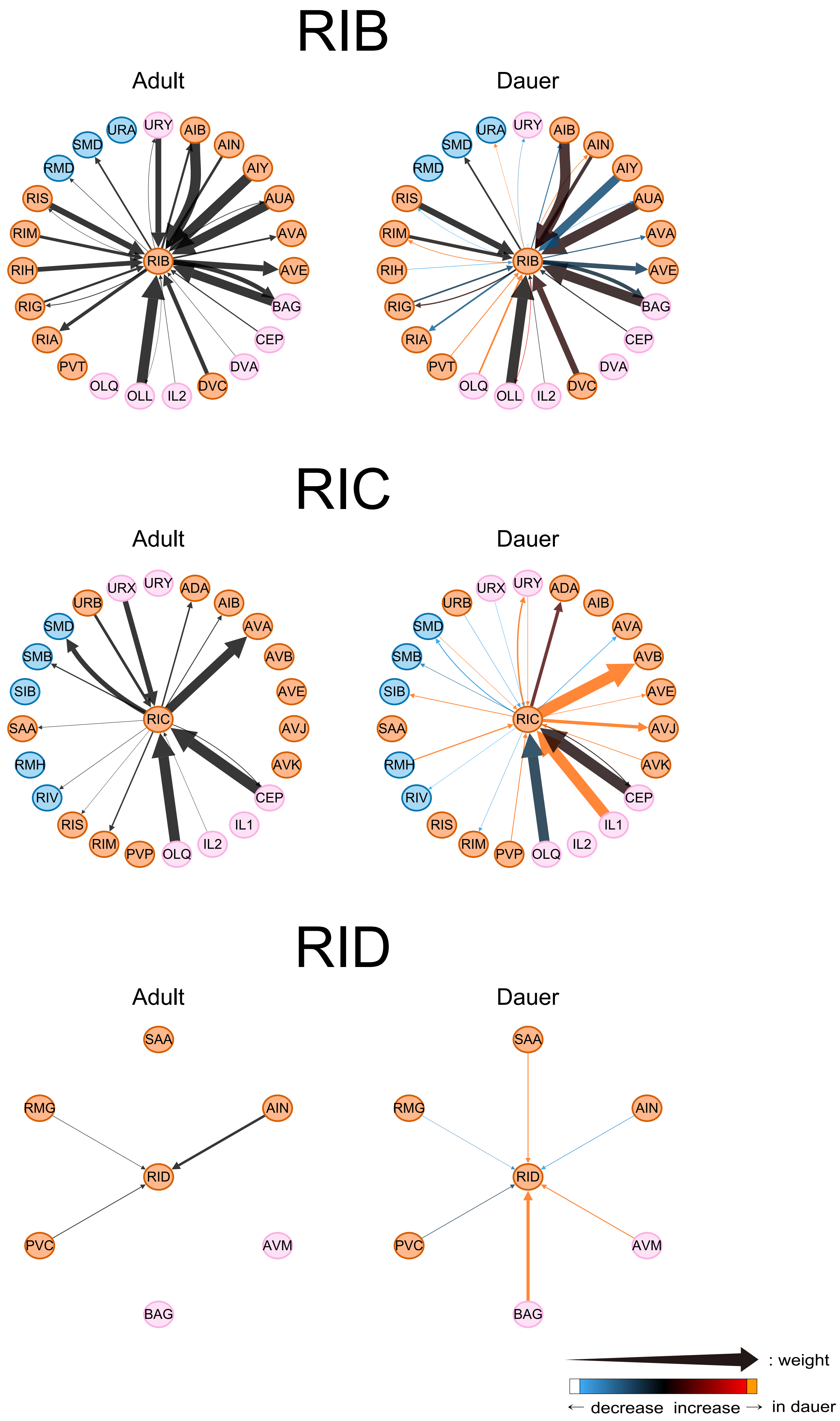
RIB
Adult
Dauer
URA
URY
AIB
AIN
AIY
AUA
AVA
AVE
BAG
CEP
DVA
DVC
IL2
OLL
OLQ
PVT
RIA
RIG
RIH
RIM
RIS
RMD
SMD
RIB
RIC
Adult
Dauer
URX
URY
ADA
AIB
AVA
AVB
AVE
AVJ
AVK
CEP
IL1
IL2
OLQ
PVP
RIM
RIS
RIV
RMH
SAA
SIB
SMB
SMD
URB
RIC
RID
Adult
Dauer
SAA
RMG
AIN
RID
PVC
AVM
BAG
: weight
← decrease increase → in dauer

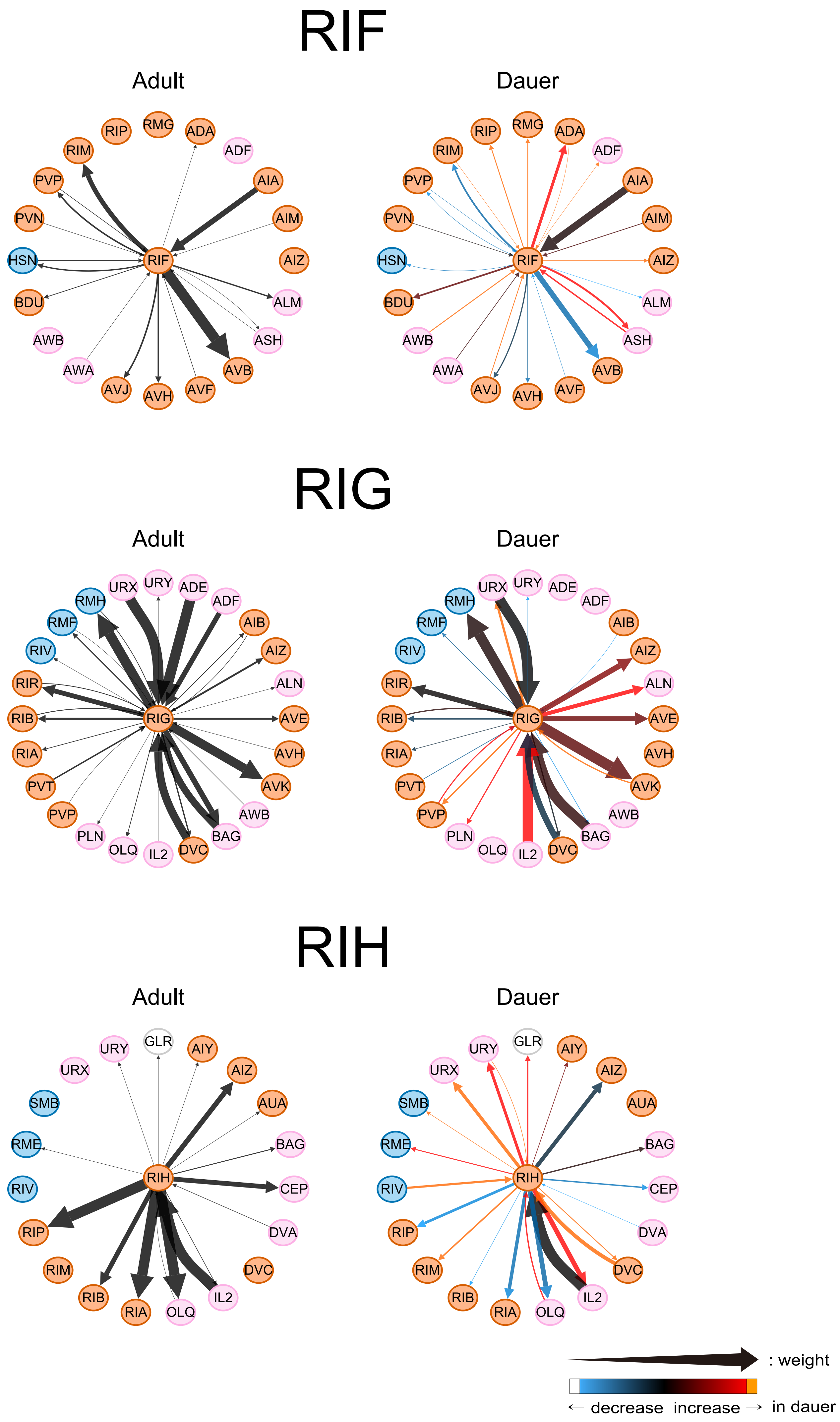
RIF
Adult
Dauer
RIP RMG ADA ADF RIM AIA PVP AIM PVN AIZ HSN RIF ALM BDU ASH AWB AVB AWA AVJ AVH AVF
RIG
Adult
Dauer
URX URY ADE ADF RMH AIB RMF AIZ RIV ALN RIR AVE RIB RIG AVH RIA AVK PVT AWB PVP BAG PLN OLQ IL2 DVC
RIH
Adult
Dauer
URY GLR AIY URX AIZ SMB AUA RME BAG RIV RIH CEP RIP DVA RIM DVC RIB IL2 RIA OLQ
: weight
← decrease increase → in dauer

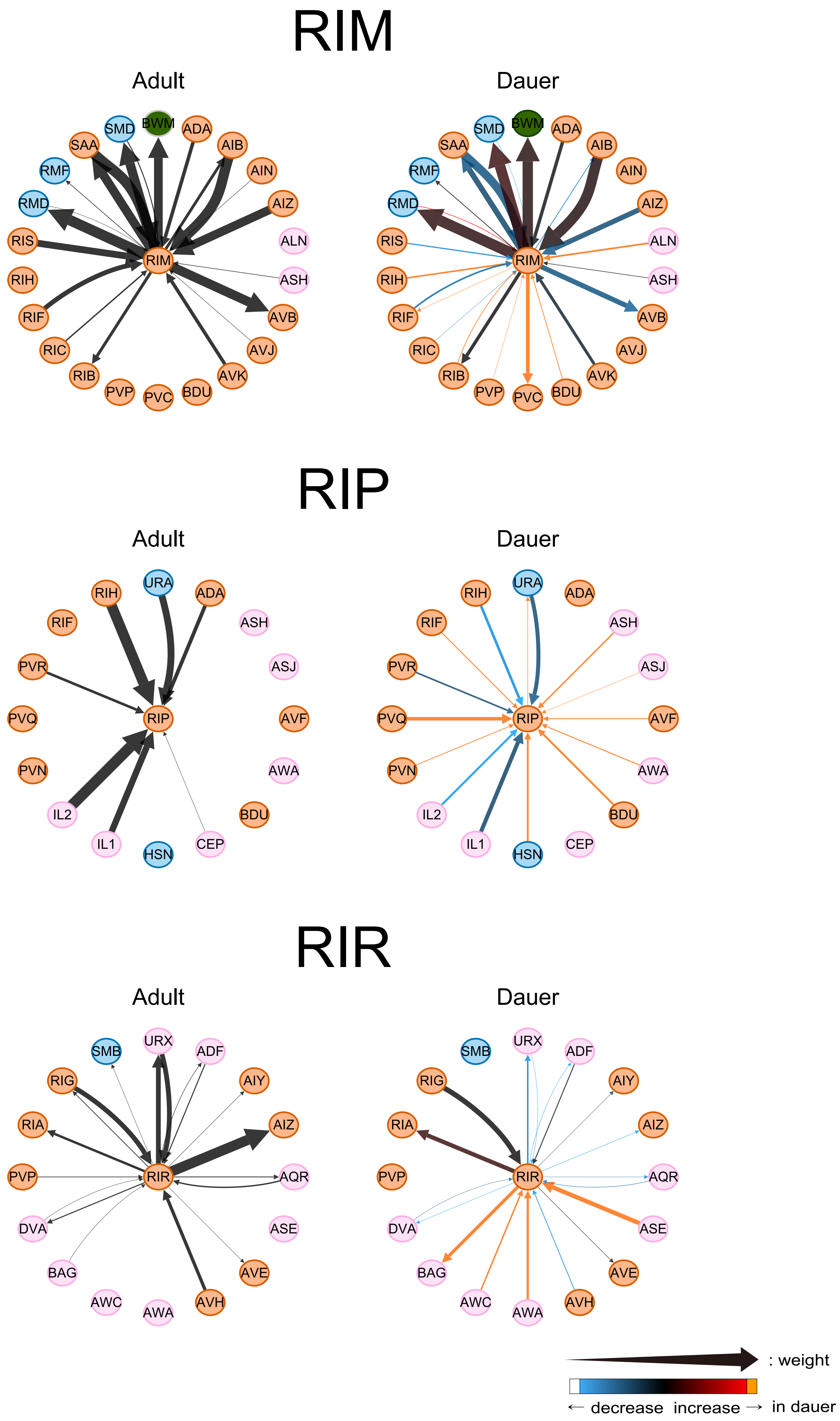
RIM
Adult
Dauer
SMD BWM ADA SAA AIB RMF AIN RMD AIZ RIS ALN RIM RIH ASH RIF AVB RIC AVJ RIB AVK PVP PVC BDU
RIP
Adult
Dauer
RIH URA ADA RIF ASH PVR ASJ PVQ RIP AVF PVN AWA IL2 BDU IL1 HSN CEP
RIR
Adult
Dauer
SMB URX ADF RIG AIY RIA AIZ PVP RIR AQR DVA ASE BAG AVE AWC AWA AVH
: weight
← decrease increase → in dauer

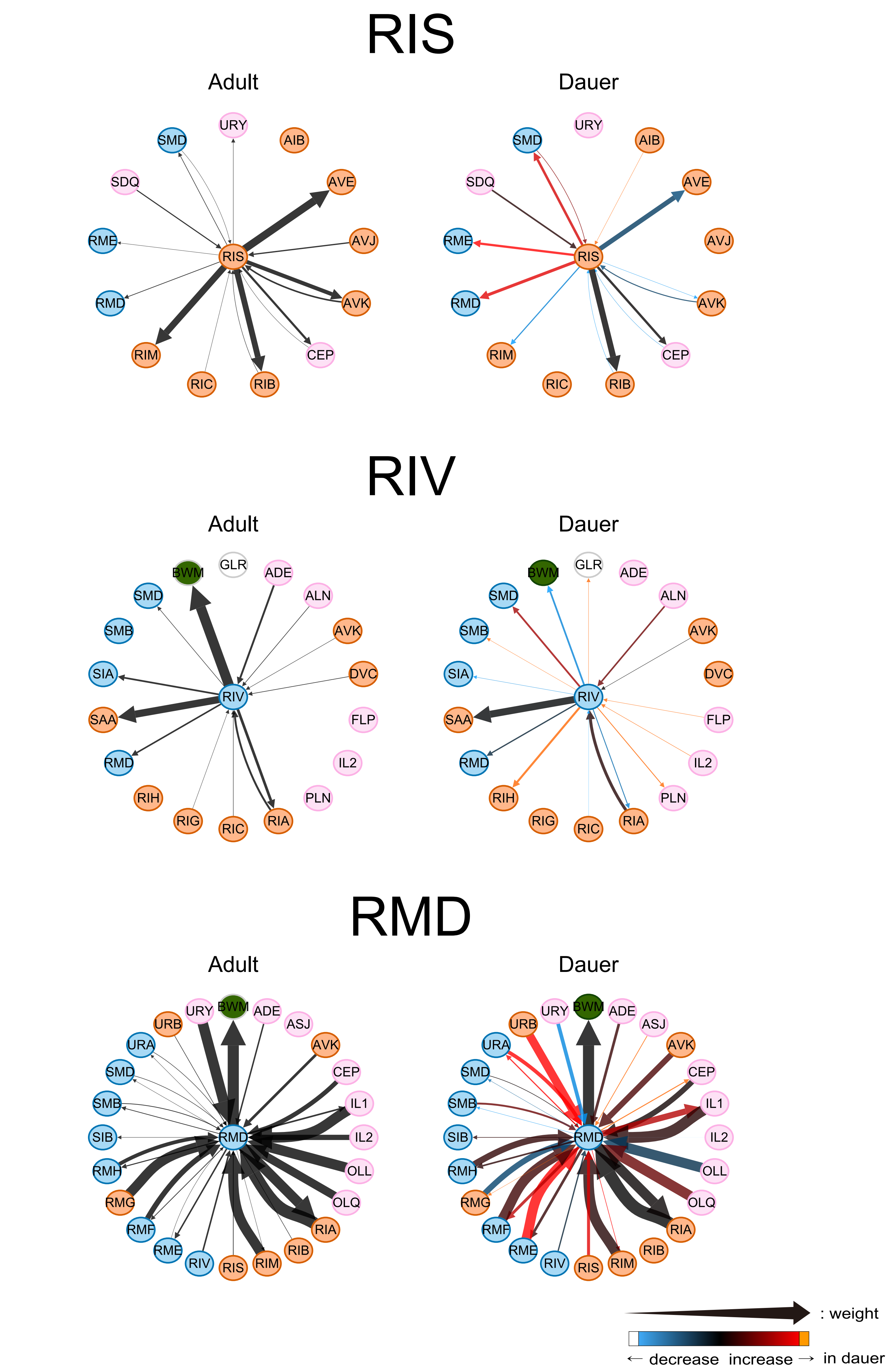
RIS
Adult
Dauer
URY
SMD
AIB
SDQ
AVE
RME
AVJ
RIS
RMD
AVK
RIM
CEP
RIC
RIB
RIV
Adult
Dauer
BWM
GLR
ADE
SMD
ALN
SMB
AVK
SIA
DVC
RIV
SAA
FLP
RMD
IL2
RIH
PLN
RIG
RIC
RIA
RMD
Adult
Dauer
URY
BWM
ADE
URB
ASJ
URA
AVK
SMD
CEP
SMB
IL1
SIB
RMD
IL2
RMH
OLL
RMG
OLQ
RMF
RIA
RME
RIB
RIV
RIS
RIM
: weight
← decrease increase → in dauer

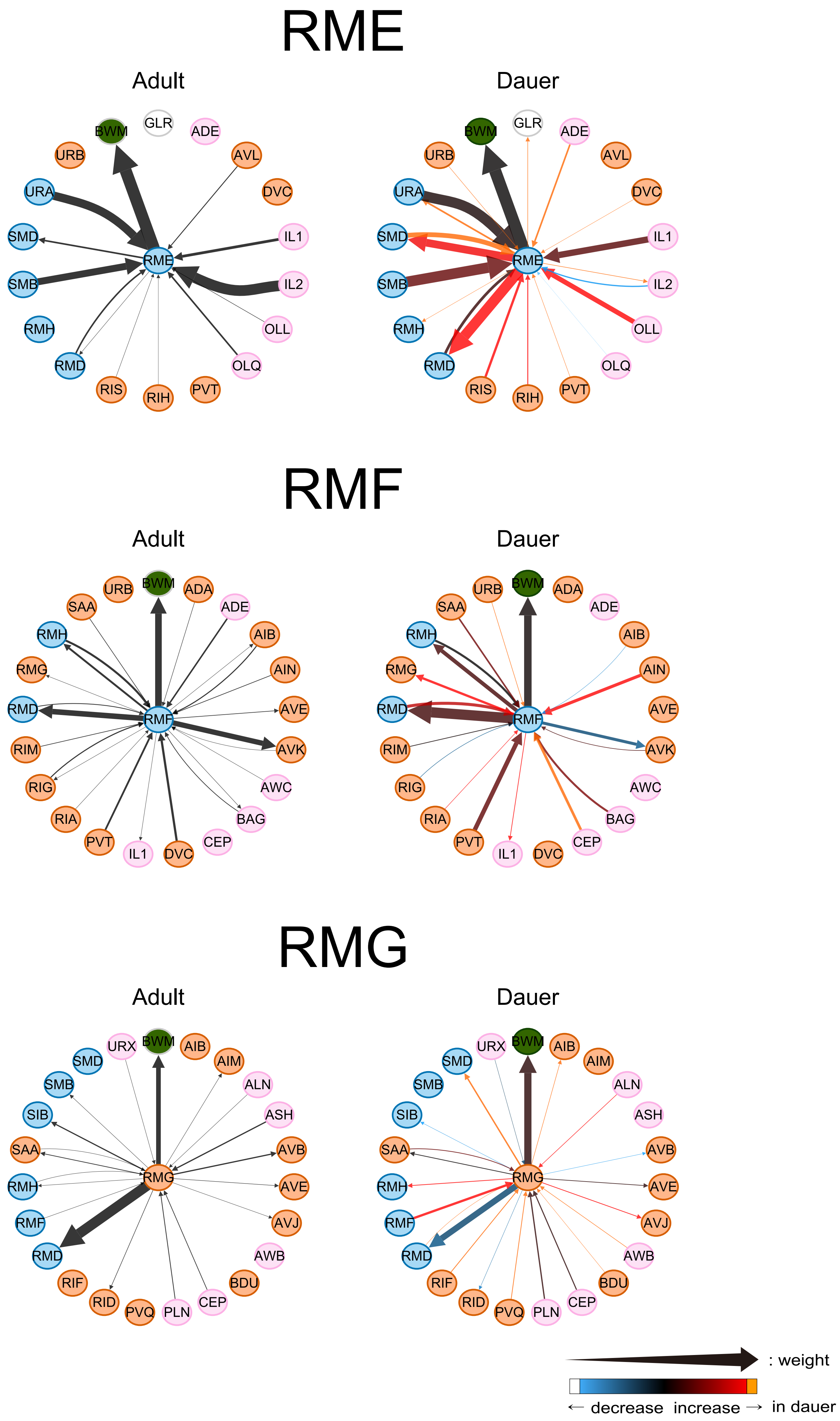
RME
Adult
Dauer
BWM GLR ADE URB AVL URA DVC SMD IL1 RME SMB IL2 RMH OLL RMD OLQ RIS RIH PVT
RMF
Adult
Dauer
URB BWM ADA SAA ADE RMH AIB RMG AIN RMD AVE RMF RIM AVK RIG AWC RIA BAG PVT CEP IL1 DVC
RMG
Adult
Dauer
URX BWM AIB SMD AIM SMB ALN SIB ASH SAA AVB RMH RMG AVE RMF AVJ RMD AWB RIF BDU RID CEP PVQ PLN
: weight
← decrease increase → in dauer

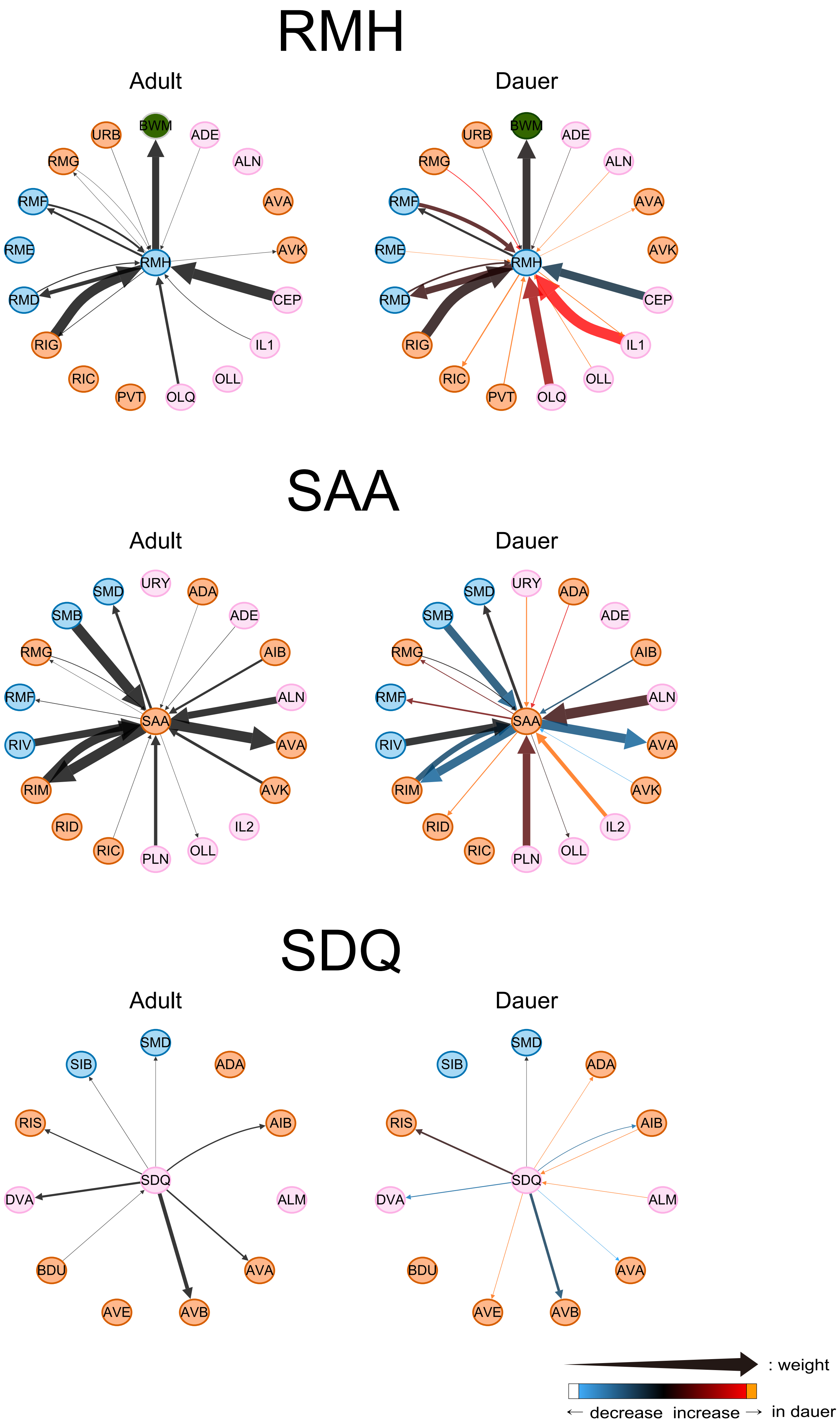

RMH
Adult
Dauer
BWM
URB
ADE
RMG
ALN
RMF
AVA
RME
AVK
RMH
RMD
CEP
RIG
IL1
RIC
OLL
PVT
OLQ
SAA
SMD
URY
ADA
SMB
ADE
RMG
AIB
RMF
ALN
RIV
AVA
RIM
AVK
RID
IL2
RIC
PLN
OLL
SDQ
SMD
SIB
ADA
RIS
AIB
DVA
ALM
BDU
AVA
AVE
AVB
: weight
← decrease increase → in dauer

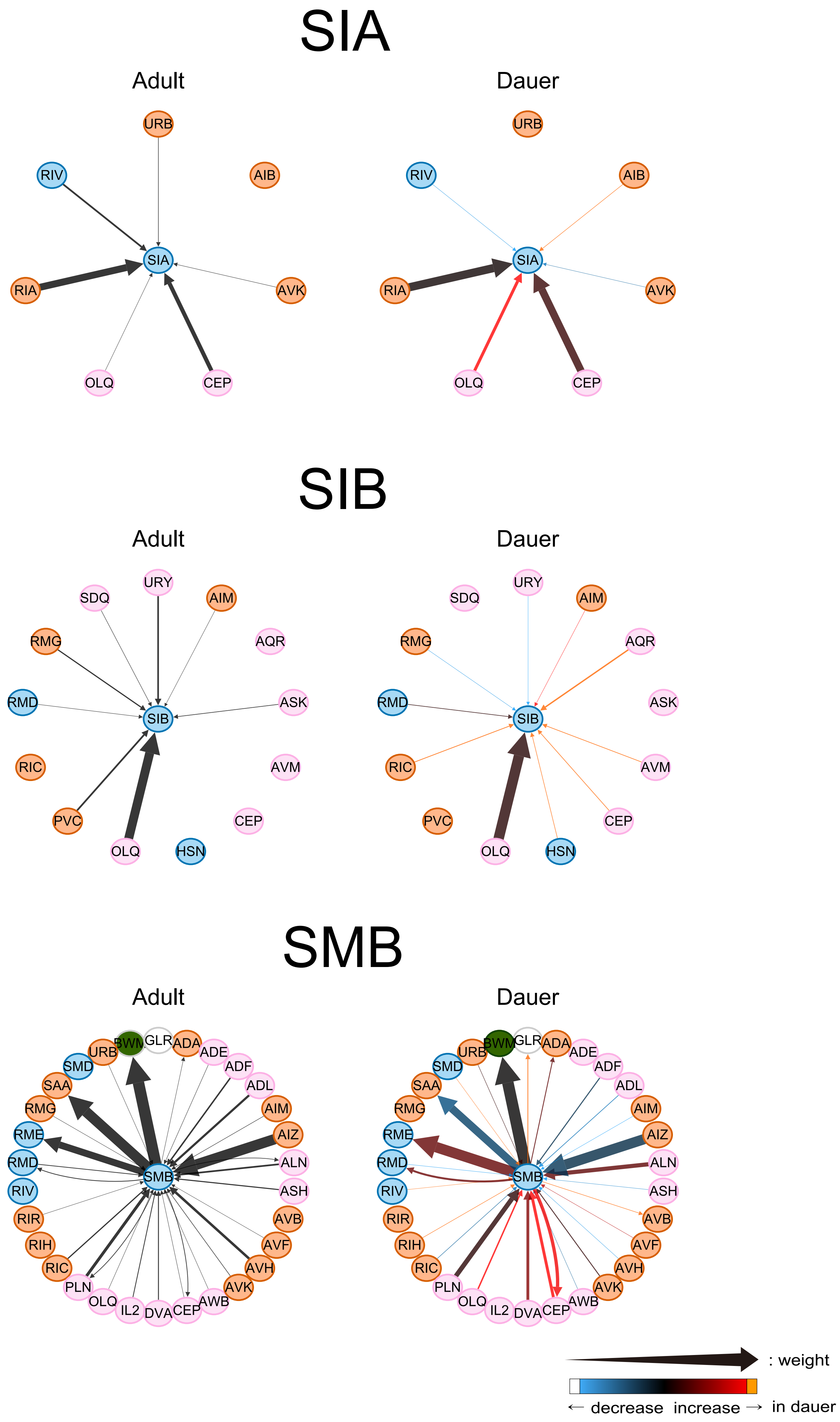
SIA
Adult
Dauer
URB
RIV
AIB
SIA
RIA
AVK
OLQ
CEP
SIB
Adult
Dauer
URY
SDQ
AIM
RMG
AQR
RMD
SIB
ASK
RIC
AVM
PVC
CEP
OLQ
HSN
SMB
Adult
Dauer
BWM
GLR
URB
ADA
ADE
SMD
ADF
SAA
ADL
RMG
AIM
RME
AIZ
RMD
ALN
SMB
RIV
ASH
RIR
AVB
RIH
AVF
RIC
AVH
PLN
AVK
OLQ
AWB
IL2
DVA
CEP
: weight
← decrease increase → in dauer

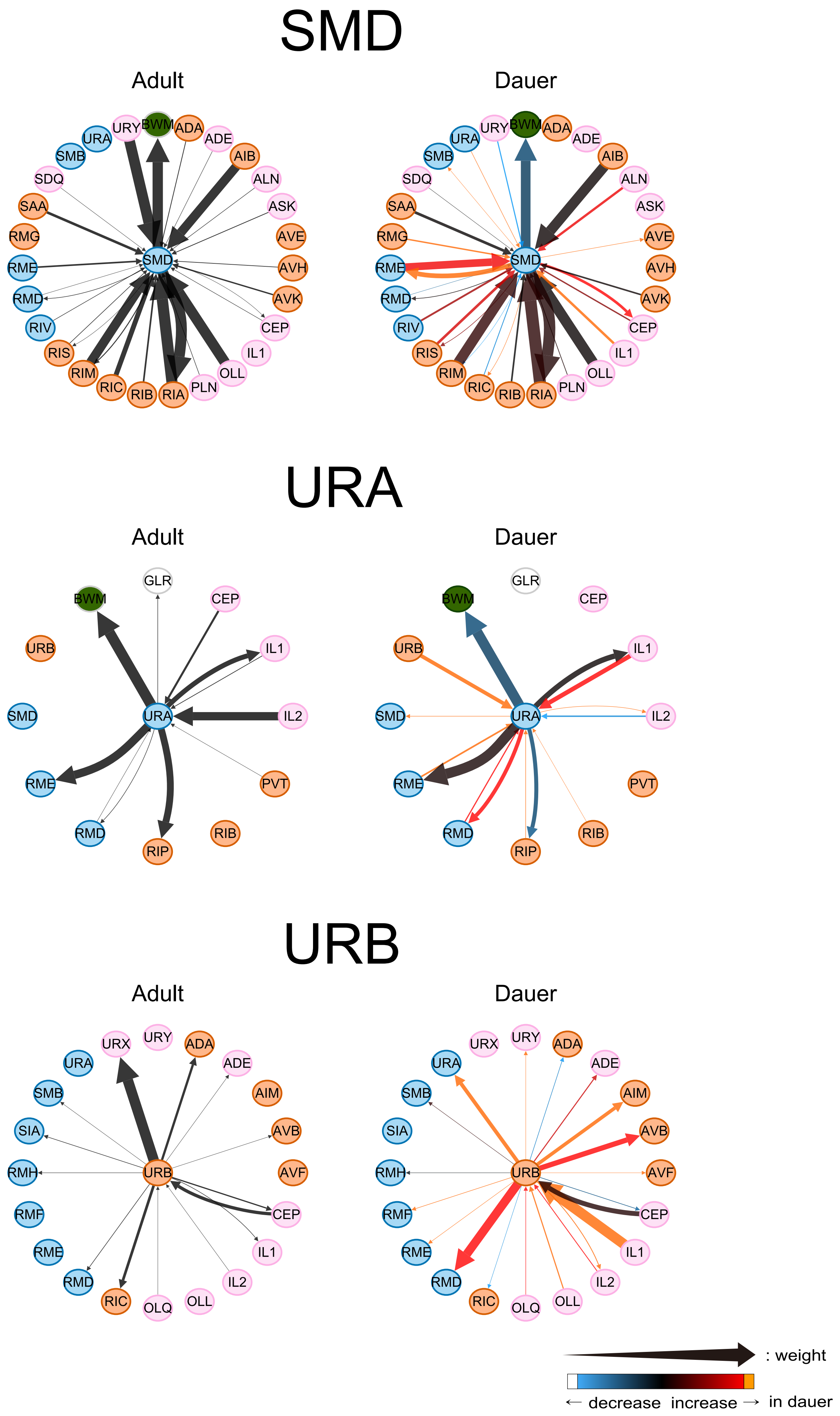
SMD
Adult
Dauer
URY BWM ADA ADE AIB ALN ASK AVE AVH AVK CEP IL1 OLL PLN RIA RIB RIC RIM RIS RIV RMD RME RMG SAA SDQ SMB URA SMD
URA
Adult
Dauer
GLR CEP IL1 IL2 PVT RIB RIP RMD RME SMD URB BWM URA
URB
Adult
Dauer
URY ADA ADE AIM AVB AVF CEP IL1 IL2 OLL OLQ RIC RMD RME RMF RMH SIA SMB URA URX URB
: weight
← decrease increase → in dauer

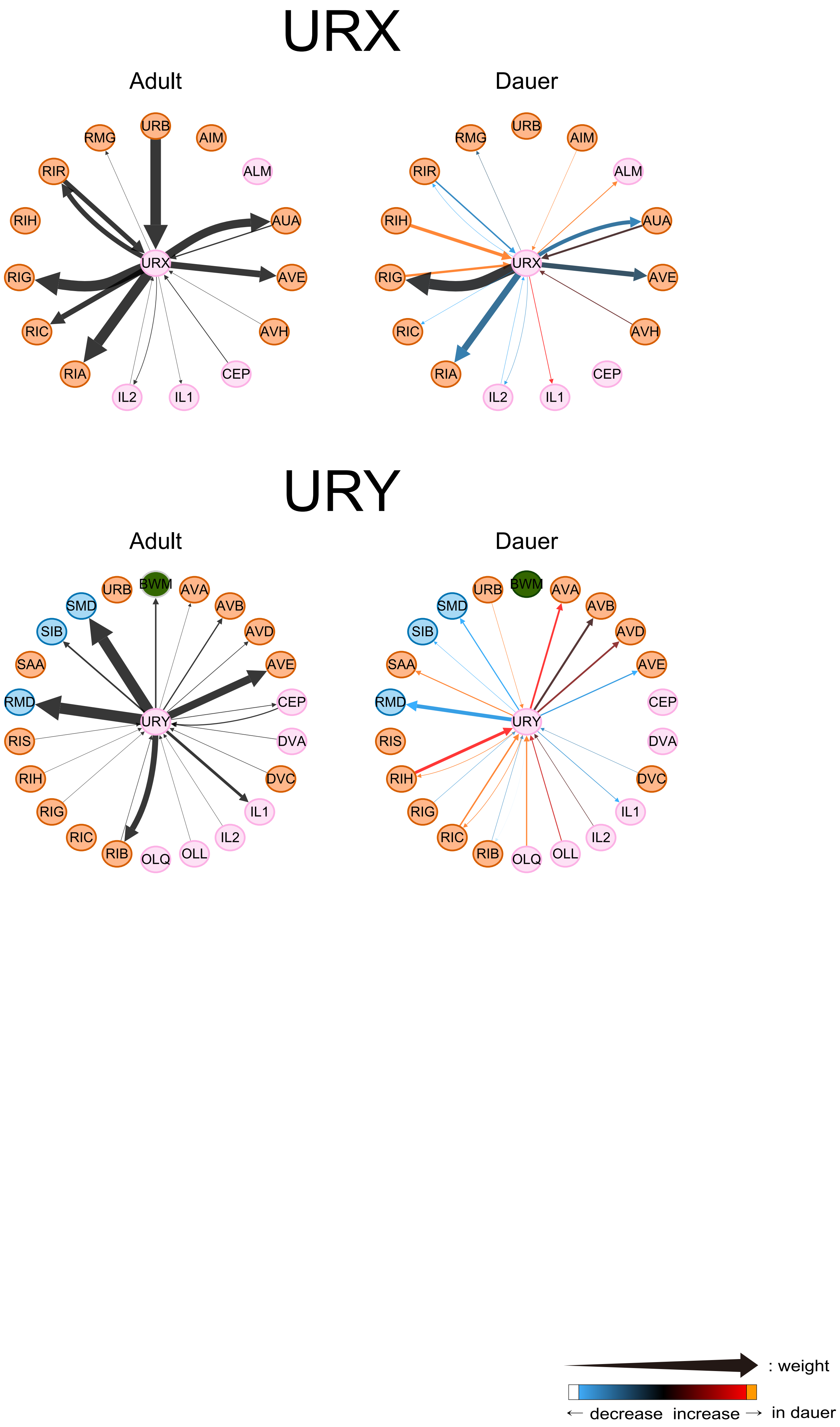
URX
Adult
Dauer
URX
URB
RMG
AIM
RIR
ALM
RIH
AUA
RIG
AVE
RIC
AVH
RIA
CEP
IL2
IL1
URY
Adult
Dauer
URY
URB
BWM
AVA
SMD
AVB
SIB
AVD
SAA
AVE
RMD
CEP
RIS
DVA
RIH
DVC
RIG
IL1
RIC
IL2
RIB
OLQ
OLL
: weight
← decrease increase → in dauer

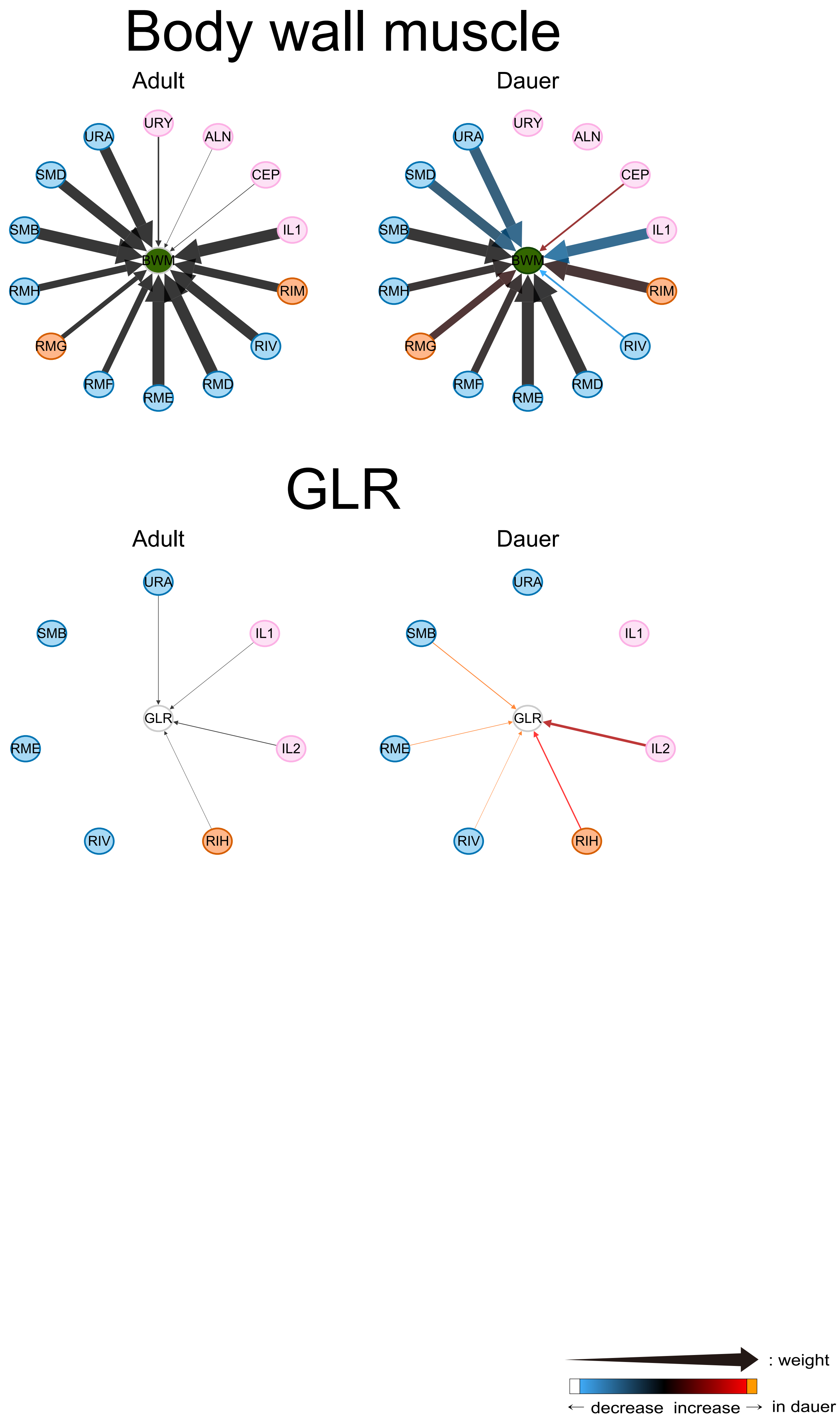

Body wall muscle
Adult
Dauer
URY
ALN
URA
SMD
CEP
SMB
IL1
BWM
RMH
RIM
RMG
RIV
RMF
RMD
RME
GLR
Adult
Dauer
URA
SMB
IL1
GLR
RME
IL2
RIV
RIH
: weight
← decrease increase → in dauer
