# Supplementary material for: Comparative connectomics of dauer reveals developmental plasticity
Source: Nat Commun. 2024 Feb 27;15:1546. doi: 10.1038/s41467-024-45943-3 (PMC10899629; doi:10.1038/s41467-024-45943-3)
Supplement: Supplementary file 10 — Reporting Summary [file 41467_2024_45943_MOESM10_ESM.pdf]

nature portfolio

Corresponding author(s): Junho Lee, Jinseop S. Kim

Last updated by author(s): Jan 17, 2024

Reporting Summary

Nature Portfolio wishes to improve the reproducibility of the work that we publish. This form provides structure for consistency and transparency in reporting. For further information on Nature Portfolio policies, see our Editorial Policies and the Editorial Policy Checklist.

Statistics

For all statistical analyses, confirm that the following items are present in the figure legend, table legend, main text, or Methods section.

n/a	Confirmed	
☐	☒	The exact sample size (*n*) for each experimental group/condition, given as a discrete number and unit of measurement
☐	☒	A statement on whether measurements were taken from distinct samples or whether the same sample was measured repeatedly
☐	☒	The statistical test(s) used AND whether they are one- or two-sided<br>*Only common tests should be described solely by name; describe more complex techniques in the Methods section.*
☐	☒	A description of all covariates tested
☐	☒	A description of any assumptions or corrections, such as tests of normality and adjustment for multiple comparisons
☐	☒	A full description of the statistical parameters including central tendency (e.g. means) or other basic estimates (e.g. regression coefficient) AND variation (e.g. standard deviation) or associated estimates of uncertainty (e.g. confidence intervals)
☐	☒	For null hypothesis testing, the test statistic (e.g. *F*, *t*, *r*) with confidence intervals, effect sizes, degrees of freedom and *P* value noted<br>*Give P values as exact values whenever suitable.*
☐	☒	For Bayesian analysis, information on the choice of priors and Markov chain Monte Carlo settings
☐	☒	For hierarchical and complex designs, identification of the appropriate level for tests and full reporting of outcomes
☒	☐	Estimates of effect sizes (e.g. Cohen's *d*, Pearson's *r*), indicating how they were calculated

Our web collection on statistics for biologists contains articles on many of the points above.

Software and code

Policy information about availability of computer code

Data collection	SerialEM 3.8 (EM imaging), TrakEM2 1.3.8 (Stitching and alignment), VAST 1.4.1 (segmentation), ZEN black edition (Carl Zeiss) (Confocal imaging)
Data analysis	Matlab R2023a, Python 3.11.4, Cytoscape 3.10.0, Cnapse (https://github.com/jabae/Cnapse), Analysis code (https://github.com/jabae/Yim-Choe-Bae-et_al-2024)

For manuscripts utilizing custom algorithms or software that are central to the research but not yet described in published literature, software must be made available to editors and reviewers. We strongly encourage code deposition in a community repository (e.g. GitHub). See the Nature Portfolio guidelines for submitting code & software for further information.

Data

Policy information about availability of data

All manuscripts must include a data availability statement. This statement should provide the following information, where applicable:

- Accession codes, unique identifiers, or web links for publicly available datasets
- A description of any restrictions on data availability
- For clinical datasets or third party data, please ensure that the statement adheres to our policy

The EM image data, volumetric cell segmentation of dauer have been deposited in BossDB and can be downloaded and viewed at https://bossdb.org/project/

yim_choe_bae2023. Synaptic connectivity data and contact data for dauer are provided as supplementary tables. The EM image data, volumetric cell segmentation, and connectivity data for other developmental stages are publicly available at https://bossdb.org/project/witvliet2020. Source data are provided with this paper.

Research involving human participants, their data, or biological material

Policy information about studies with human participants or human data. See also policy information about sex, gender (identity/presentation), and sexual orientation and race, ethnicity and racism.

Reporting on sex and gender	n/a
Reporting on race, ethnicity, or other socially relevant groupings	n/a
Population characteristics	n/a
Recruitment	n/a
Ethics oversight	n/a

Note that full information on the approval of the study protocol must also be provided in the manuscript.

Field-specific reporting

Please select the one below that is the best fit for your research. If you are not sure, read the appropriate sections before making your selection.

☒ Life sciences ☐ Behavioural & social sciences ☐ Ecological, evolutionary & environmental sciences

For a reference copy of the document with all sections, see nature.com/documents/nr-reporting-summary-flat.pdf

Life sciences study design

All studies must disclose on these points even when the disclosure is negative.

Sample size	One C. elegans dauer has been imaged and analyzed for this study. No statistical methods were used to calculate the sample size. For behavior experiments, sample size was determined based on prior studies (e.g., Lee et al., 2011) which conducted similar experiments.
Data exclusions	For connectivity analysis, neurons without any connection have been excluded as they are not valid for analysis. For analysis using clustering coefficient, neurons with less than two degrees were omitted as it is not possible to define a triangle. Please refer to figure legends and methods for details.
Replication	Data from multiple experiments were pooled, unless stated otherwise in figure legends.
Randomization	Experimental groups for behavioral assays were selected randomly.
Blinding	Investigators were blinded to the test subjects and controls during imaging and behavioral assays.

Reporting for specific materials, systems and methods

We require information from authors about some types of materials, experimental systems and methods used in many studies. Here, indicate whether each material, system or method listed is relevant to your study. If you are not sure if a list item applies to your research, read the appropriate section before selecting a response.

Materials & experimental systems

n/a	Involved in the study	
☒	☐	Antibodies
☒	☐	Eukaryotic cell lines
☒	☐	Palaeontology and archaeology
☐	☒	Animals and other organisms
☒	☐	Clinical data
☒	☐	Dual use research of concern
☒	☐	Plants

Methods

n/a	Involved in the study	
☒	☐	ChIP-seq
☒	☐	Flow cytometry
☒	☐	MRI-based neuroimaging

Animals and other research organisms

Policy information about studies involving animals; ARRIVE guidelines recommended for reporting animal research, and Sex and Gender in Research

Laboratory animals	Caenorhabditis elegans (N2 Bristol strains and other transgenics). Animals used for EM imaging and behavior experiments were 1-day dauer. Animals used for confocal imaging were L3, 1-day adult, 1-day dauer, and 1-day postdauer adult.
Wild animals	No wild animals were used in this study.
Reporting on sex	All animals were hermaphrodites.
Field-collected samples	No field collected samples were used in this study.
Ethics oversight	This study did not require an ethical approval.

Note that full information on the approval of the study protocol must also be provided in the manuscript.

Plants

Seed stocks	n/a
Novel plant genotypes	n/a
Authentication	n/a
